# Interventions for improving the health and socio-economic well-being of young parents: a scoping review of studies in Africa

**DOI:** 10.1136/bmjopen-2026-117430

**Published:** 2026-09-25

**Authors:** Grace Kathure Mugo, Glorious Jesang, Sally Atieno Odunga, Beatrice W Maina, Caroline W Kabiru, Anthony Idowu Ajayi

**Affiliations:** 1 African Population and Health Research Center, Nairobi, Kenya; 2 University of Ghana, Accra, Ghana; 3 Egerton University, Njoro, Kenya

**Keywords:** Africa, Health, Adolescent, Parents

## Abstract

**Abstract:**

**Objectives:**

Early childbearing has significant adverse effects on the health and socio-economic well-being of young people globally. While some interventions have addressed the challenges faced by young parents, no study has systematically mapped initiatives aimed at improving the health and socio-economic well-being of young parents in Africa. This scoping review aimed to map and summarise existing interventions meant to improve the well-being of young parents in Africa, examining their approaches, geographical distribution, key outcomes and impacts to inform future policy and programme decisions.

**Design:**

Our study adhered to the Preferred Reporting Items for Systematic Reviews and Meta-Analyses extension for Scoping Reviews (PRISMA-ScR) standards and was guided by the Joanna Briggs Institute methodological framework for scoping review.

**Data sources:**

We conducted a systematic search across PubMed, Science Direct, JSTOR, African Journals Online, Education Resources Information Center and Google Scholar for peer-reviewed articles published between 1 January 2000 and 31 December 2024.

**Eligibility criteria for selecting studies:**

We included peer-reviewed studies such as randomised controlled trials, non-randomised studies, mixed methods studies and qualitative evaluations focusing on young mothers and fathers aged 10–24 years in Africa, reporting interventions targeting their health and socio-economic well-being and published in English or French. We excluded non-peer-reviewed studies, studies conducted outside of Africa, those focusing on parents older than 24 years, publications written in languages other than English or French as well as grey literature materials.

**Data extraction and synthesis:**

Three independent reviewers screened all articles’ abstracts and titles using Rayyan software. Then these three reviewers independently conducted a full-text review for the prequalified articles using the prespecified inclusion criteria. Findings were summarised in tables and synthesised qualitatively.

**Results:**

Our review included 13 peer-reviewed studies, namely randomised controlled trials (2), quasi-experimental (7), mixed-methods (2) and qualitative studies (2). The studies were conducted in seven African countries, primarily in Kenya (5) and exclusively targeted young mothers (10–24 years), while neglecting young fathers. Most interventions focused on health outcomes, particularly mental and sexual and reproductive health. Few addressed economic (financial independence, livelihoods), social (communication, decision-making) or educational (school continuation, performance) outcomes. The interventions demonstrated positive impacts across multiple domains, including mental health, education, sexual/reproductive health access, social skills (communication, decision-making) and economic empowerment (financial independence, living conditions) among young mothers.

**Conclusion:**

The evidence base for interventions targeting African young parents’ well-being is limited in volume, geographical spread, evaluation rigour and focus on young fathers. The findings underscore the need for more robust research to identify effective interventions for this population.

**PROSPERO registration number:**

CRD42023464828.

**STRENGTHS AND LIMITATIONS OF THIS STUDY**

- Our study was guided by the Joanna Briggs Institute methodological framework for scoping reviews, ensuring rigorous and systematic mapping of evidence.
- However, we may have missed interventions that have not been published in peer-reviewed literature, as well as those published in languages other than English or French.
- The diversity of intervention designs and outcome measures precluded a conclusive assessment of effectiveness.
- The findings should therefore be read as preliminary signals of potential positive effects and warrant confirmation in more homogeneous or adequately powered studies.

## Introduction

 Adolescent childbearing (births between ages 10 and 19) remains a global phenomenon, with higher rates being reported in developing countries and some Global North countries such as the UK and USA.^1^ Globally, approximately 13% of girls begin childbearing before turning 18 years of age.^2 3^ According to a systematic review on prevalence and determinants of adolescent pregnancy in Africa, adolescent fertility stood at 18.8%, with East Africa recording the highest prevalence (21.5%) and the lowest in Northern Africa (9.2%).^4^ Adolescent childbearing contributes to increased school dropouts, which consequently impacts educational attainment and human capital development.^5 6^ Because of the lower educational attainment, girls who become pregnant in their adolescence are less likely to secure well-paying jobs to meet their needs, including their parental obligations.^7^ This often leads to an intergenerational cycle of poverty, though some of the young parents overcome this challenge and lead productive lives. A study by the World Bank in 2011 revealed that if all young women and adolescent girls in Kenya completed their basic education and all young mothers were engaged in an income-generating activity instead of experiencing early childbirth, it would contribute to a boost in the country’s economy by raising an additional US$3.4 billion annually.^8^ Early childbearing continues to undermine the socio-economic well-being of adolescent girls as well as the national functioning through lower labour force participation and attenuated educational attainments.^9^

Girls who become pregnant are often stigmatised due to social, cultural and religious beliefs against premarital sex.^10^ This results in hostile home environments, which may force girls to leave their natal homes.^11^ For some of these girls, marriage or cohabitation with the fathers of their children becomes the only socially acceptable path to restoring their social status and receiving financial support to cater to their needs and those of their children.^12^ However, early marriage or cohabitation exposes young mothers to mental distress,^13^ school dropout^14^ and domestic violence.^15^ It also increases their risk of a rapid repeat pregnancy.^16^

Though previous studies have indicated that the effects of early childbearing are more direct, manifest and grave on young mothers compared with young fathers,^17^ the few studies that have focused on young fatherhood have revealed that early paternity is not without socio-economic consequences for the father himself.^18^ Emerging research shows that early fatherhood can have similarly harmful effects, including poor physical and psychosocial health, school dropout, financial challenges and substance use and abuse.^19–21^ Early fatherhood affects the educational outcomes of young fathers and becomes an economic burden to themselves and the community at large.^19 22^ Young fathers are also at a heightened risk of engaging in substance abuse and experiencing mental health problems.^23^

The intergenerational cycle of early parenthood has been well documented among young mothers.^24–28^ Daughters born to young mothers have a greater likelihood of becoming young mothers compared with daughters of older mothers.^24–26^ This intergenerational effect has also been observed among young fathers, with evidence also showing that sons born to young fathers were 1.8 times more likely to become fathers in their early adulthood compared with sons born to older fathers.^29^ These repeated early births contribute to poverty and disadvantages within families and societies.^30^

Breaking the intergenerational cycle of early parenthood and its adverse effects requires gender transformative, multisectoral interventions that empower young parents, build their resilience and improve their overall well-being. Past studies on young parenthood in Africa have documented lived experiences and recommended interventions to enable young parents to successfully navigate parenthood and improve their overall health and socio-economic well-being.^31–33^ While some studies have implemented interventions towards this goal, limited research has systematically mapped the nature of extant interventions and their effectiveness.^34 35^ Existing reviews of interventions targeting young parents have only focused on psychosocial interventions^36–38^ and interventions seeking to avert repeat births.^39 40^ Often, these reviews fail to include studies focused on Africa, despite the region having the highest adolescent pregnancy prevalence. While the state of evidence on interventions to improve the health and well-being of young parents is nascent, mapping the few existing studies could help inform future studies and interventions. Considering the often intertwined socio-economic and cultural factors that shape the experiences of young mothers and fathers in Africa, this scoping review aimed at mapping and summarising existing interventions meant to improve the well-being of young parents in Africa, examining their approaches, geographical distribution, key outcomes and impacts to inform future policy and programmatic decisions. More specifically, this review sought to address this research question: What are the existing interventions that have been implemented to improve the health and socio-economic well-being of young parents in Africa?

## Methods

### Study design

Our study adhered to the Preferred Reporting Items for Systematic Review and Meta-analyses extension for Scoping Reviews (PRISMA-ScR) standards and was guided by Joanna Briggs Institute (JBI) methodological framework for scoping reviews.^41^ This review followed five of the six JBI stages namely (1) identification of the research question; (2) identification of relevant studies; (3) selection of eligible studies; (4) charting the data; and (5) collating, summarising and reporting of the results.^42^ Stage six of the JBI framework, which involves consulting stakeholders, is optional. However, we informally consulted with various colleagues working in relevant areas as the review progressed through the five steps. Our study’s protocol was registered with the International Prospective Register of Systematic Reviews (PROSPERO) at the University of York, UK, under the registration number CRD42023464828^43^ (see online supplemental file 1). All studies included in the review met the prespecified eligibility criteria outlined in the protocol^43^ and methodological adaptations appropriate for a scoping review. The study was initially conceived as a systematic review; hence, the protocol published is a systematic review protocol. However, since our review question was broad and sought to understand the existing body of evidence on interventions targeting young parents in Africa, which resulted in diverse interventions and outcomes identified in the existing evidence,^44^ a scoping review was a more appropriate approach. Thus, a scoping review was conducted to map the available evidence on the interventions aiming at improving the health and socio-economic well-being of young parents in Africa.

### Review question

The general objective of this scoping review was to map and summarise the existing interventions aimed at improving the well-being of young parents in Africa. The specific research questions were as follows:

1. What are the interventions that have been implemented to enhance the health and socio-economic well-being of young parents in Africa?
2. What are the geographical distribution and the approaches that have been used to implement these interventions?
3. What are the outcomes of these interventions related to the health and socio-economic well-being of young parents in Africa?
4. What are the effects of these interventions on the health and socio-economic well-being of young parents in Africa?

### Search strategy

Our search was guided by the JBI methodological framework for scoping reviews Population, Concept and Context (PCC) framework as shown in table 1. The population element of the PCC framework refers to the population of interest, the concept element is the main theme or the topic of interest that the review aims to explore, and the context captures the settings under which the population and concept elements are situated.^41^ We searched African Journals Online, JSTOR, Education Resources Information Center, PubMed, Google Scholar and Science Direct for published peer-reviewed articles focused on interventions to improve the health and socio-economic well-being of young parents in Africa (see online supplemental file 2). Additionally, we screened the reference lists of peer-reviewed articles to identify additional articles that could be incorporated into this review. Our study was limited to studies that were conducted in Africa only. We focused on studies that were published between 1 January 2000 and 31 December 2024 on young parents aged 10–24 years.

**Table 1:** PCC framework for eligibility of studies included in the scoping review

Framework item	Components
Population	Young parents aged 10–24 years in Africa.
Concept	Interventions/programmes/projects targeting or including young parents, such as peer-led interventions, young mothers’ clubs, community-based interventions, support groups, parenting programmes Health and socio-economic well-being. Health (mental health or psychosocial well-being, repeat pregnancy, sexual and reproductive health knowledge, transactional sex, lifestyle diseases, postnatal care, nutrition, family planning or contraception). Socio-economic well-being (education, skill acquisition, financial literacy skills, parenting skills, socio-economic empowerment).
Context	Africa/African Nations

Our study employed various search terms, which we structured into four key blocks, namely interventions, young parents (aged 10–24 years), Africa and health and socio-economic well-being. We included all the African countries in the search to capture different interventions that have been implemented in different African nations. We adopted search terms accordingly for every bibliographic database in combination with specific filters. Our study employed Boolean operators, including (‘AND’, ‘OR’ and ‘NOT’), to narrow or expand the search specificity. We used dates, language (French and English) and age as the key restrictions on our search criteria. We present a supplementary document to demonstrate our search strategies in the six databases (see online supplemental file 1).

### Inclusion and exclusion criteria

We included studies that: (1) focus on young mothers and/or fathers aged 10–24 years or belonging to a broader age range, but presented separate results for young mothers and/or young fathers aged 10–24 years; (2) examined interventions targeting young parents in Africa, regardless of whether their children or pregnant adolescents were also targeted in the same study; (3) were either published in English or French; (4) were published between 1 January 2000 and 31 December 2024; (5) reported health and socio-economic outcomes; and (6) peer-reviewed studies.

We excluded articles that: (1) only reported on interventions targeting pregnant mothers (10–24 years old); (2) articles that reported on interventions that targeted children of young parents and not the parents themselves; (3) studies that focused on improving young mothers’ pregnancy outcomes, such as studies that tested interventions to improve antenatal care attendance; (4) studies that are not peer-reviewed and (5) studies not implemented in Africa.

### Study selection

We used three steps to identify and select articles that met our inclusion criteria. In the first step, we removed all duplicates that appeared in our initial search results. Second, three independent reviewers (GJ, GKM and SAO) screened all articles’ abstracts and titles using Rayyan software. Lastly, three reviewers (GJ, GKM and SAO) independently conducted a full-text article review for the prequalified articles using the prespecified inclusion criteria. At this stage, the articles that were agreed on by the reviewers were included in the final list, and any disagreements at any stage were resolved through a discussion with all the authors of this study.

### Data extraction

We used a standardised Microsoft Excel form to extract data from our included studies (see online supplemental file 3). Reviewers independently extracted data to ensure consistency and uniformity of the data extracted in the final extraction. We specifically extracted information including the title of the study, study publication year, study authors, region, subregion, country(ies) where the interventions were implemented, study sample size, participants’ age bracket, sex of the participants involved in the study, as well as specific methodology information such as design of the study, the type of data and objectives of the study. Additionally, the name and type of interventions, whether they fell under socio-economic and health well-being of young parents in Africa, were extracted to identify which interventions focused on specific facets. Two reviewers (GJ and GKM) independently extracted the information from all the included studies, and one reviewer (SAO) crosschecked the information extracted to ensure reliability.

### Data analysis and synthesis

We processed our extracted data and organised it into tables and figures. This allowed us to summarise the key interventions identified, the participants’ demographic characteristics and the geographical distribution of our work within Africa. We extracted the interventions from the studies and grouped them as either socio-economic or health interventions. Our synthesis also included information on whether the young parents were involved in co-creating the interventions or not. We employed a qualitative approach in our analysis, given that our objective was to map and describe the interventions that have been implemented to improve the health and socio-economic well-being of young parents in Africa. We employed a thematic analysis approach to pinpoint significant patterns that were notable in our data extracted from the qualitative studies. We created codes and categorised the codes based on our study’s objective and the significant data elements that informed the coding strategy. We used Microsoft Excel software to code. Two reviewers checked the variations, similarities and differences that came up in the results.

### Deviations from the study protocol

Initially, we intended to conduct a systematic review using both peer-reviewed studies and grey literature to capture the evidence of possible interventions being implemented among young parents in Africa. However, our review question was broad, and the search yielded diverse interventions with a wide range of outcomes, so a systematic review could not be possible which resulted in the change from systematic review to a scoping review. We also excluded grey literature (see online supplemental file 4) due to concerns about the quality of the evidence presented given the lack of external review.

### Patient and public involvement

There were no patients involved in our study.

## Results

Our initial search across targeted databases and snowball references yielded 1371 records after all filters were applied. We retrieved 1045 articles with titles and abstracts from which we removed 478 duplicates. We screened 567 of the remaining articles and excluded 487 articles that did not meet our predefined inclusion criteria. We assessed a total of 80 full-text papers and excluded 67 peer-reviewed articles that did not meet our inclusion criteria, leaving us with a total of 13 peer-reviewed articles for our analysis (see figure 1).

**Figure 1:**
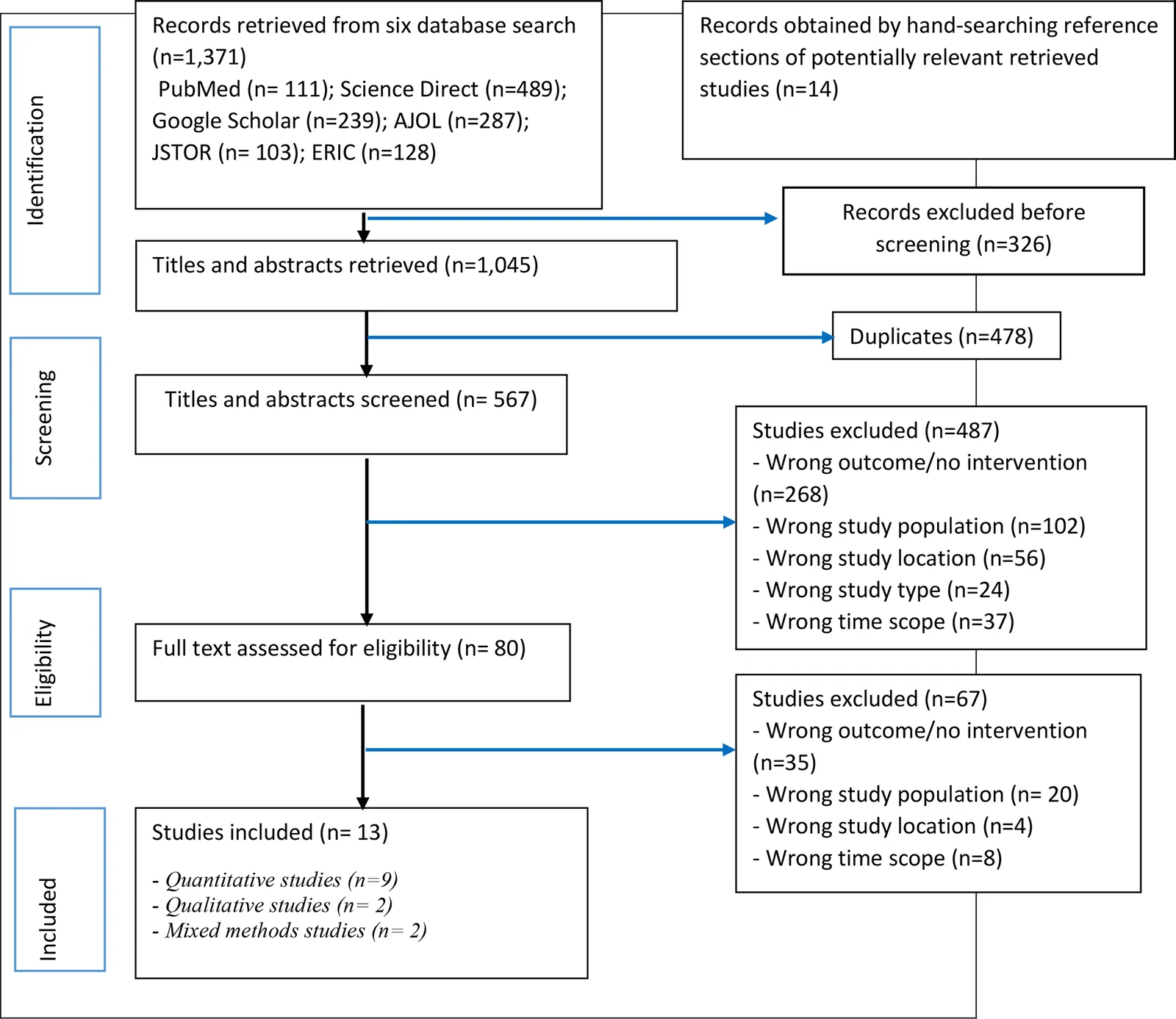
PRISMA-ScR flow diagram detailing the search and selection process. AJOL, African Journals Online; ERIC, Education Resources Information Center; JSTOR; PRISMA-ScR, Preferred Reporting Items for Systematic Reviews and Meta-Analyses Extension for Scoping Reviews.

### Characteristics of included studies

A total of 13 intervention studies published in peer-reviewed articles met the inclusion criteria. Two of the 13 peer-reviewed articles reported on randomised controlled trials,^45 46^ 7 on non-randomised quantitative studies or quasi-experimental studies,^47–53^ 2 on mixed methods studies^54 55^ and 2 on qualitative studies^56 57^ (see table 2).

**Table 2:** Characteristics of included studies

Author	Country	Study design	Intervention	Comparison
Berry *et al*^53^	South Africa	Quasi-experimental study with intervention and control groups	Intervention group participated in a parenting programme meant to improve their parenting skills and well-being.	Comparison group did not receive parenting programme package.
Gage *et al*^51^	DRC	Quasi-experimental using a pretest-post-test comparison design	Intervention group received an integrated family planning, maternal and newborn health and nutrition intervention.	Comparison group received standard care.
Yator *et al*^45^	Kenya	Randomised controlled trial (pilot study)	Intervention group received IPT-G.	Waitlist control+usual PMTCT care (Prevention of Mother-to-Child Transmission services).
Gureje *et al*^46^	Nigeria	Cluster randomised controlled trial	Intervention package comprising behavioural activation and problem-solving treatment, parent skills training and social and parenting skills support provided by a ‘neighborhood mother’.	Control group received usual care at the primary maternal care clinics.
Levy *et al*^47^	Kenya	Quasi-experimental study with before-and-after comparisons among the same group of non-randomised participants at both time points	Intervention group participated in a home visiting programme of pregnant adolescent girls, adolescent mothers and their children/infants.	Comparison group is the same group of non-randomised participants interviewed at baseline and endline.
Mhlanga *et al*^52^	Zimbabwe	Before-and-after descriptive cross-sectional study design, quasi-experimental study	Intervention group received a social behaviour change mobilisation package on sexual and reproductive health outcomes.	Comparison group is the same group of participants at endline.
Mwenda *et al*^48^	Kenya	Quasi-experimental with a control group at endline	Two intervention groups, with one receiving a limited package (interactive text messaging only) and the full package (text messaging and weekly support groups, moderated by a community health extension worker and counsellor.	Comparison group received usual care and was recruited at endline.
Opondo *et al*^49^	Kenya	Quasi-experimental, ex post facto causal-comparative research design	Intervention group comprised of teenage mothers enrolled back in school who received guidance and counselling.	Comparison group comprised of teenage mothers who enrolled back in school without receiving any guidance and counselling.
Tinago *et al*^50^	Zimbabwe	Quasi-experimental with two study arms	Community-based peer support interventions to eliminate social isolation and stigma among adolescent mothers.	Comparison group was adolescent mothers living in different communities, 35 km from the intervention community, with similar characteristics. The control arm received a US$5 gift card after completing baseline, midline and endline surveys.
Kachingwe *et al*^55^	Malawi	Mixed-methods studies	Intervention group received training on various aspects of their psychosocial life for 2 years.	Comparison group received psychosocial training during the second year of the implementation of the intervention.
Chingono *et al*^54^	Zimbabwe	Mixed-methods study	Self-help group intervention package comprising 12 participatory sessions where adolescent mothers were trained on healthy parenting, developing individual and group problem-solving skills, importance of self-care, safer sexual practices and appropriate health-seeking, as well as capacity to manage and save money.	Comparison group comprised the same group of participants at endline.
Leerlooijer *et al*^56^	Uganda	Qualitative evaluation	Teenage mothers project comprising community awareness raising, teenage support groups, livelihood empowerment, counselling and advocacy.	Teenage mothers who had participated in the project at endline.
Yator *et al*^57^	Kenya	Qualitative evaluation	IPT-G for postpartum adolescent mothers living with HIV.	Waitlist group who received the IPT-G intervention from week 20.

DRC, Democratic Republic of Congo; IPT-G, Group Interpersonal Psychotherapy.

### Geographical distribution of studies

The included studies were conducted in seven countries in Africa, with the majority (5) of them being in Kenya,^45 47–49 57^ followed by three studies in Zimbabwe^50 52 54^ and one each in Malawi,^55^ Uganda,^56^ Nigeria,^46^ South Africa^53^ and the Democratic Republic of Congo (DRC)^51^ (see figure 2).

**Figure 2:**
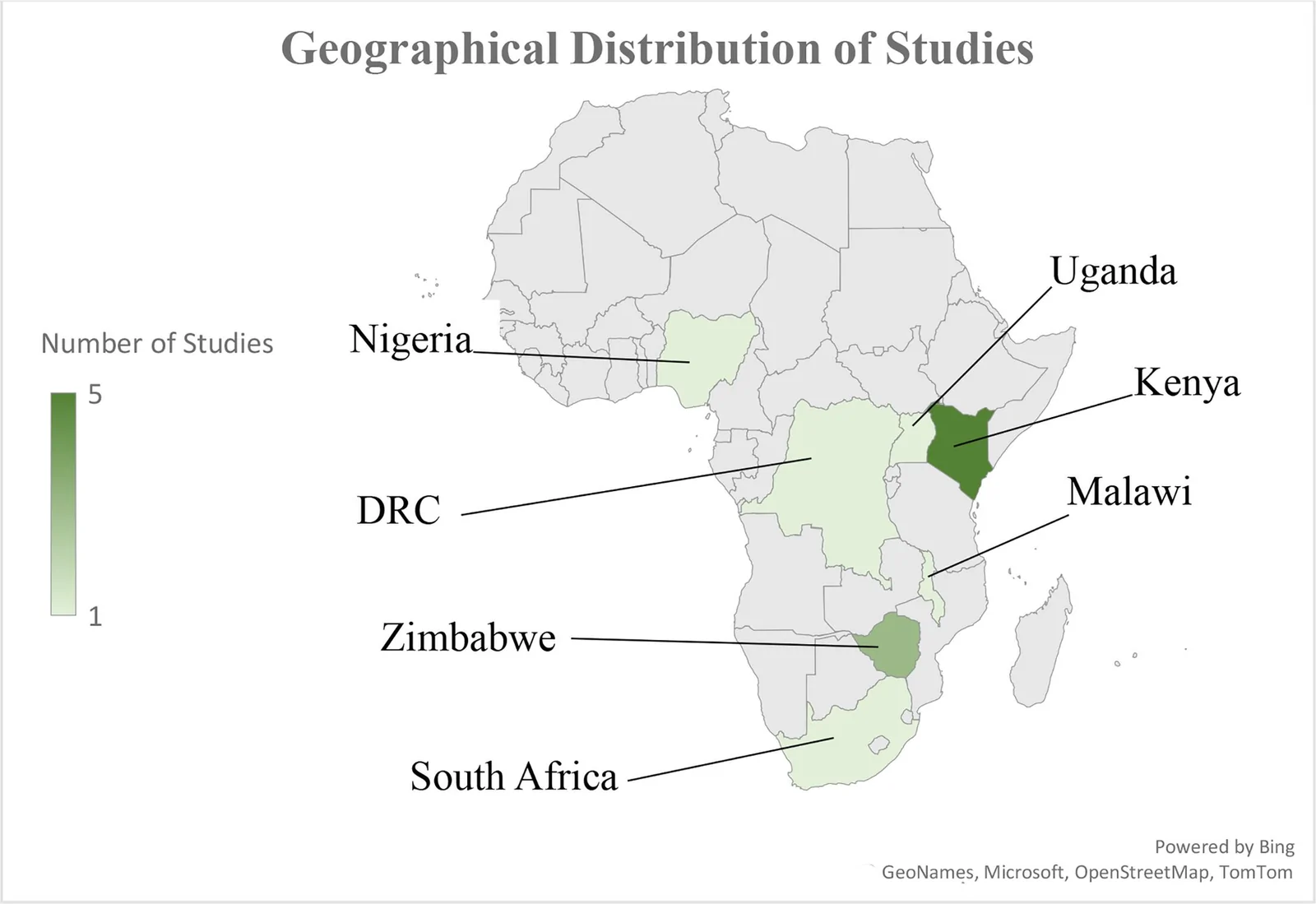
Geographical distribution of studies.DRC, Democratic Republic of Congo.

### Target population and involvement of young parents in the design and implementation of the intervention

Some of these interventions included young mothers alone,^46 50 51 53–55 57^ while others targeted young mothers and pregnant adolescents^47 48^ or young mothers and their infants.^48 55^ One intervention targeted young mothers and their male partners.^51^ One intervention targeted both young parents and adolescents with parental responsibilities for a non-biological child.^53^ The interventions included young mothers of different age groups, ranging between 10 years and 24 years. There were fewer interventions focused on young mothers younger than 15 years^48 56 57^ compared with those involving young mothers older than 15 years. Some of the studies implemented interventions targeting young mothers who were HIV-positive.^45 47 57^ Only three studies reported interventions involving young mothers in the design and implementation of the interventions.^46 47 56^ The other 10 studies did not specify whether they involved young parents or not (see table 3), and no study reported involving young fathers in co-designing and implementation of interventions. Ways in which the young mothers were involved included being consulted through meetings and discussions during the design of interventions,^46 56^ and implementing the interventions as mentors to other young mothers after being trained.^47^

**Table 3:** Target groups and involvement of young parents in intervention design and/or implementation

Author/implementer	Country	Target group	Involvement of young parents in the intervention design and/or implementation
Berry *et al*^53^	South Africa	Biological (n=20) and non-biological (n=93) adolescent parents aged between 12 and 22 years from three secondary schools in Cape Town, South Africa; 61 assigned to the intervention group and 52 to the control group. All the biological parents were included in the intervention group.	Not specified
Chingono *et al*^54^	Zimbabwe	Adolescent girls aged 16–19 years (n=93), residing in the selected communities, reported selling sex as their main means of earning a livelihood and were either recently pregnant or had at least one child up to 24 months old.	Not specified
Gage *et al*^51^	DRC	Married and unmarried adolescent and young first-time mothers aged 15–24 years (n=1927) and their male partners.	Not indicated
Gureje *et al*^46^	Nigeria	Pregnant adolescents aged <20 years at fetal gestational age 16–36 weeks with moderate to severe depression and parenting adolescent mothers (n=242; intervention arm, n=141; control arm, n=101).	Yes
Kachingwe *et al*^55^	Malawi	Adolescent mothers aged 18 years (n=211) and their children.	Not specified
Leerlooijer *et al*^56^	Uganda	Nine former unmarried teenage mothers were selected from the teenage mothers who had ever participated in the Teen Mothers project.	Yes
Levy *et al*^47^	Kenya	HIV-vulnerable and HIV-positive pregnant adolescent girls (n=384) and mothers (n=203) aged 10–19 years.	Yes
Mhlanga *et al*^52^	Zimbabwe	Pregnant adolescents and adolescent mothers from two clinics (n=60, with 30 from each clinic) in the Epworth District of Zimbabwe, 10–19 years.	Not specified
Mwenda *et al*^48^	Kenya	Adolescent mothers aged younger than 20 years (n=1011) and their infants who are younger than 3 months. The intervention group had 791 adolescent mother-infant dyads (416 received a limited package and 375 received the full package); 220 dyads were assigned to the control group.	Not specified
Opondo *et al*^49^	Kenya	Teenage mothers enrolled back in school who went through a guidance and counselling programme (n=138) and teenage mothers integrated back into the school without going through the counselling programme (n=104).	Not specified
Tinago *et al*^50^	Zimbabwe	Adolescent mothers aged 15–18 years (n=183), with 104 assigned to the intervention group and 79 to the control group.	Not specified
Yator *et al*^57^	Kenya	Postpartum adolescents aged 15–24 years, comprising an intervention group (n=13) and a waitlist group (n=12) living with HIV.	Not specified
Yator *et al*^45^	Kenya	Postpartum young women aged 18–25 who were 6–12 weeks postpartum, had HIV and attended a ‘preventing mother-to-child transmission’ programme (n=24).	Not specified

DRC, Democratic Republic of Congo.

### Intervention settings and duration

Most interventions took place in group settings within hospital or school facilities, community settings and using peer supporters^46 51 52 54^ (see table 4). Other interventions were administered to individual young mothers.^47 48^ Additionally, some interventions were delivered to both groups and individuals.^48 56^ All these interventions were carried out through face-to-face interactions. Few interventions were implemented over longer periods of time, exceeding 12 months,^51 55^ while the majority of other interventions were carried out over relatively short durations, with eight conducted within a period of less than 12 months.^46 48 49 51 54–57^

**Table 4:** Intervention duration and delivery

Author/implementer	Country	Approach	Delivery settings	Duration	Frequency
Berry *et al*^53^	South Africa	Group sessions	Group sessions per week that included role plays and small discussions facilitated by programme facilitators	20 weeks	Weekly group sessions
Chingono *et al*^54^	Zimbabwe	Group sessions	10-15 in self-help groups	6 months	12 participatory sessions
Gage *et al*^51^	DRC	Individual, group sessions and community-based approach	Home visits Group education sessions Social and behaviour change communication activities targeting the community and significant influencers of FTMs	16 months	Monthly home visits Monthly group education sessions Behaviour change activities, thrice per month
Gureje *et al*^46^	Nigeria	Individual sessions	Mobile phone calls and texts (as appropriate	Six sessions during the antenatal period	Weekly sessions and three fortnightly sessions
Kachingwe *et al*^55^	Malawi	Group sessions	Dialogue sessions at YWCA safe spaces	2 years	Two dialogue sessions a month, lasting 4 hours
Leerlooijer *et al*^56^	Uganda	Individual and group sessions	Community	March 2000, March 2012	Not specified
Levy *et al*^47^	Kenya	Individual sessions	Home visits, engagement with community members	6 months	Not specified
Mhlanga *et al*^52^	Zimbabwe	Group sessions	Community health centres	6 months	Weekly sessions lasting 2 hours per session
Mwenda *et al*^48^	Kenya	Individual and group	Messaging and group settings	9 months	Weekly
Opondo *et al*^49^	Kenya	Not specified	Not specified	Not specified	Not specified
Tinago *et al*^50^	Zimbabwe	Group sessions	Community	6 Months	12 sessions
Yator *et al*^57^	Kenya	Group sessions	Primary health facilities	6 months	Weekly sessions
Yator *et al*^45^	Kenya	Group setting	Health facilities	16 weeks with the intervention group and 8 weeks with the control group	Weekly sessions

DRC, Democratic Republic of Congo; FTMs, first-time mothers; YWCA, Young Women's Christian Association.

### Intervention category by outcomes

This scoping review focused on both health and socio-economic outcomes, including educational and economic outcomes. The majority of the studies (8) reported on two outcomes.^47 48 50 52 54–57^ Three reported on one outcome^46 49 51^ and one study on three outcomes^53^ (see table 5). Eight studies reported on an intervention aimed at enhancing the health and socio-economic outcomes of the target population^46–49 51 52 56 57^ while nine reported on interventions meant to improve socio-economic outcomes.^47 48 50 52–57^ Further, nine studies reported on interventions aimed at improving social outcomes such as parenting skills, parent–child communication and resilience.^47 48 50 52–57^ Two studies reported on interventions that aimed to improve the educational outcomes of young mothers by facilitating their school re-entry and academic adjustment,^49 56^ and two studies reported on interventions that aimed to improve the economic outcomes of the young mothers.^54 56^ While only one study captured an intervention targeting the male partners of these young mothers,^51^ no specific outcomes were reported for these male partners (see online supplemental file 5 for detailed summary of interventions and their effects).

**Table 5:** Summary of interventions targeting young parents

Author/implementer	Country	Description of intervention	Outcome(s)		Effects of the interventions
			Health	Social, economic and educational outcomes	
Berry *et al*^53^	South Africa	Adolescent parenting programme adopted a group-based approach with two focus areas, namely developing positive parenting skills and supporting adolescent development for responsible adulthood. The study further assessed whether depression influences parenting outcomes.	Depression	Parenting skills and resilience	Improved positive parenting and increased individual resilience.
Chingono *et al*^54^	Zimbabwe	Self-help groups led by a trained facilitator on problem-solving skills, practice-based learning for healthy parenting and safer sexual practices, the importance of self-care and appropriate health-seeking, and improving capacity to manage and save money.	Mental well-being	Self-confidence Saving and investing money	Enhanced mental health well-being and socio-economic outcomes.
Gage *et al*^51^	DRC	Momentum Project interventions offered integrated family planning, maternal and child health, as well as nutrition activities.	Contraceptive uptake Mother and child health Nutrition	Contraceptive use norms, behaviour and personal agency	Improved postpartum family planning behaviour
Gureje *et al*^46^	Nigeria	Behavioural activation and problem-solving treatment, parenting skills training and social and parenting skills support provided by a self-identified adult ‘neighborhood mother’.	Depressive symptoms	Parenting skills	Lower levels of depressive symptoms and better parenting practices.
Kachingwe *et al*^55^	Malawi	Provision of safe and inclusive community spaces for adolescent mothers and pregnant girls.	Nutritional practice Motor skills and cognitive functions	Parenting skills Socioemotional capacities	Better parenting skills and improved nutritional practice, motor skills and cognitive functions.
Leerlooijer *et al*^56^	Uganda	Teenage mother project with five components namely: community sensitisation, teenage mothers support groups, livelihood (continued education and income generation), counselling and advocacy.	Mental well-being through improved self-confidence and self-worth and coping skills with adolescent motherhood and stigma	Community support, Increased agency, Continuation of schooling Increased earnings among teenagers	Supportive community norms towards teenage mothers’ position and future opportunities, increased agency, improved coping with early motherhood and stigma, continued education through returning to school after pregnancy and increased income generation by teenage mothers.
Levy *et al*^47^	Kenya	Home visiting programme by mentors (who were young mothers aged 19–30 years).	Modern family planning, viral load suppression and increase in ART uptake	Household support	Enhanced household support, increased uptake of modern contraceptive methods and ART uptake.
Mhlanga *et al*^52^	Zimbabwe	Peer-based approach called ‘Champions of Change’, which involved establishing targeted peer support groups for pregnant adolescents and adolescent mothers.	Sexual and reproductive health knowledge	Parental attitudes toward adolescents’ access to sexual and reproductive health services	Increased access to adolescent-friendly SRHR services, an increase in the number of adolescents with a positive attitude towards the use of long-acting reversible contraceptives and an increase in the number of adolescents reporting improved attitudes on SRHR among parents.
Mwenda *et al*^48^	Kenya	Targeted messages on childcare and nurturing and psychosocial support groups for adolescent mothers, moderated by community extension workers and counsellors.	Not indicated	Maternal knowledge of childcare and infant development milestones and exclusive breastfeeding	Increased maternal knowledge of child care and infant development milestones.
Opondo *et al*^49^	Kenya	School guidance and counselling programme.	Not indicated	Social and academic adjustment of teenage mothers	Better academic adjustment for those who went through formal guidance and counselling before enrolling back in school.
Tinago *et al*^50^	Zimbabwe	Community-based peer support programme aimed at mitigating social isolation and stigma among adolescent mothers.	Depressive symptoms and common mental disorders	Household and friends’ support	Decreased probability of moderate to severe depression and increased household and friends’ support.
Yator *et al*^57^	Kenya	Group interpersonal therapy intervention (IPT-G) delivered by community health workers.	Depressive symptoms	Social functioning	Notable improvements in social interaction, decision-making and communication skills as a result of the intervention.
Yator *et al*^45^	Kenya	IPT-G.	Depressive symptoms ART adherence	No information provided	Reduction in depressive symptoms.

ART, antiretroviral therapy; DRC, Democratic Republic of Congo; IPT-G, Group Interpersonal Psychotherapy; SRHR, sexual and reproductive health and rights.

#### Health outcomes

Of the 13 studies that reported interventions aimed at improving health outcomes, 7 focused on enhancing the mental health of young parents^46 47 51 52 54–56^; 3 targeted improvements in sexual and reproductive health (SRH) knowledge and access to SRH services^47 51 52^; and 1 aimed to increase HIV counselling and testing, viral load suppression and antiretroviral therapy (ART) adherence^45^ (see table 5). Four studies reported on interventions to improve mental health outcomes using psychosocial support groups,^50 54–56^ two adopted the Group Interpersonal Psychotherapy (IPT-G) approach,^45 57^ and three studies reported on interventions that provided individual mental health counselling and/or treatment sessions.^46 49 56^

The psychosocial support groups included sessions with young mothers on problem-solving skills, the importance of self-care, building resilience and self-esteem, and coping with early motherhood and stigma.^50 54–56^ On the other hand, IPT-G involved therapy sessions delivered in groups, where young mothers learnt from one another. They discussed common life challenges and suggested practical solutions among themselves.^45 57^ Individual mental health counselling and/or treatment sessions consisted of counselling each young mother on their personal life challenges and how to cope with them,^49 56^ and during the treatment, each young mother was guided through a step-by-step process of breaking down their current psychosocial stressors and exploring solutions.^46^

Out of the four studies that used psychosocial support groups, three reported an improvement in the mental health and well-being of young mothers,^50 54 56^ as shown in table 5. An intervention implemented in Zimbabwe by Chingono *et al* reported that the psychosocial groups led to adolescent mothers having a sense of hope, feeling their mental well-being improved due to the new social networks and increased confidence in seeking health services for their babies and themselves.^54^ Chingono *et al*^54^ also reported a decline in signs of common mental disorders among those in the psychosocial group from 16.0% to 2.0% at follow-up. Similarly, another study conducted in Uganda found that psychological support enhanced adolescent mothers’ sense of dignity, confidence and self-worth and also enabled them to cope effectively with their challenges.^56^ On the other hand, Kachingwe *et al* did not find a statistically significant difference in confidence and psychosocial well-being of adolescent mothers, which they attributed to the multidimensional nature of the psychosocial element.^55^ Specifically, they noted that psychological well-being was linked to many parameters including poverty and limited life purpose suggesting that such programmes should always consider incorporating all dimensions of psychosocial well-being.

Studies that used the IPT-G approach^45 57^ reported a reduction in depressive symptoms among young mothers after the intervention administration in both the intervention and control groups. Young mothers who participated in IPT-G interventions reported significant improvements in peer interaction, communication skills and decision-making.^57^ They also gained confidence in discussing their life challenges.

Similarly, individual mental health counselling and/or treatment interventions were also reported to have led to better mental health outcomes for young mothers.^46 49 56^ Young mothers who received counselling generally showed better emotional and psychological adjustments when they returned to school after delivery of their children compared with those who had not received counselling prior to enrolling back to school.^49^ Further, counselling was reported to have led to increased agency and improved coping with early motherhood challenges and stigma among young mothers.^56^ According to Gureje *et al* young mothers who had undergone mental health counselling and treatment had lower levels of depressive symptoms.^46^

Only three studies reported on interventions focusing on the provision of essential SRH information and services to young mothers.^47 51 52^ The information and services included contraceptive services and comprehensive sexuality education. One of the studies by Levy *et al* reported that provision of SRH information and services to young mothers improved the uptake of modern contraceptives and empowered them to make informed choices about their SRH.^47^

An intervention on childcare and nurturing by Mwenda *et al* found that compared with the control group, teenage mothers receiving the intervention had a higher knowledge score on infant care and development (9.02 vs 8.01; p<0.001), higher exclusive breastfeeding rates (63.5% vs 50.9%; p=0.004) and their infants had higher average Developmental Milestones Checklist (DMC III) scores (53.09 vs 48.59; p=0.01)^48^ (see online supplemental file 5). Another study reported on an intervention that aimed to improve viral load suppression whereby a home visiting programme by young mother mentors led to an increase in ART uptake and viral load suppression among young mothers living with HIV^47^ (see table 5).

#### Social, economic and educational outcomes

In Uganda, a study by Leerlooijer *et al* reported on an intervention that aimed to create a supportive social environment for teenage mothers through community sensitisation and advocacy.^56^ The intervention contributed to more supportive community norms toward adolescent mothers’ future opportunities, including continuing education and pursuing income-generating activities. However, Leerlooijer and colleagues also reported little change in disapproving community norms regarding premarital sex and pregnancies.^56^ They noted that support extended to young mothers was interpreted by some community members as tacit approval of premarital sex and early pregnancy, pointing to the need for greater sensitivity to prevailing social norms when designing and implementing interventions.^56^ Levy and colleagues^47^ reported a significant increase in household support following a household visit intervention implemented in Kenya. The percentage of families demonstrating this support—meaning the willingness and actions of the caregiver(s) or partner to actively engage in and support the adolescent’s health, well-being and future opportunities—rose from 57% to 85% during the programme.^47^

Three studies out of the 13 studies focused on interventions aimed at enhancing the parenting skills and resilience of the young mothers.^46 53 55^ For example, Gureje *et al* reported that an intervention package on parenting skills training and the involvement of a ‘neighborhood mother’—an experienced older woman willing to support and guide the adolescent mothers in performing their parental role, led to improved parenting skills.^46^ An adolescent parenting programme implemented in South Africa using a group-based approach resulted in an improvement in positive parenting skills and resilience among young mothers in both the treatment and comparison groups.^53^

Only 2 studies out of the 13 studies addressed interventions aimed at enhancing the economic outcomes of young mothers through vocational training, entrepreneurial and leadership skills training^54 56^ (see table 5). Young mothers who participated in a teenage mother’s empowerment project implemented in Uganda reported increased financial independence and improved living conditions due to greater employment opportunities and other opportunities for sustainable income generation.^56^

In addition, some adolescent mothers who participated in the IPT-G treatment meant to enhance the social functioning of postpartum adolescents in Kenya through communication and self-awareness trainings/capacity building reported that they had set up small businesses with significant improvement in their communication skills and interaction with their peers.^57^ Adolescent mothers who received counselling were able to continue with education and generally demonstrated better academic adjustments by performing well on returning to school, compared with those who did not receive counselling.^49 56^

## Discussion

This review study systematically mapped interventions aimed at improving the health and socio-economic well-being of young parents in Africa. We found that existing interventions are limited in both geographical scope and volume, with only 13 studies reporting interventions identified overall. Most studies focused on just 7 out of Africa’s 54 countries, with close to half of these studies (5) capturing interventions implemented in Kenya.^45 47–49 57^ It is striking that almost half of these studies have been conducted in a single East African country—a region with a comparatively lower burden of teenage pregnancy—while the most affected regions, such as Central and West Africa, have seen relatively few studies. The concentration of interventions implemented in Kenya may reflect subnational disparities within the country, where certain counties experience disproportionately high adolescent pregnancy rates,^58^ warranting localised interventions and sustained attention.

Our review indicates that interventions captured in the included studies overwhelmingly targeted young mothers, with only one intervention including male partners^51^ and without clarifying whether these were young fathers. This points to the invisibility of young fathers in research and policy in Africa. This maternal-centric focus may reflect entrenched gender norms that position child-rearing as the mother’s responsibility, while overlooking the role of fathers in supporting maternal and child health. Engaging young fathers more intentionally could enhance family well-being, strengthen parenting practices and promote more equitable outcomes. Future interventions should therefore move beyond mother-only approaches and explicitly include young fathers.

Notably, only 3 of the 13 studies reported involvement of young mothers in intervention design and implementation, mainly through co-development and serving as mentor mothers.^46 47 56^ Lack of involvement of young parents in intervention design and implementation is a missed opportunity to ground programmes in the lived realities of their intended beneficiaries. It also means many interventions may not have fully captured the perspectives and priorities of young parents, potentially limiting their resonance and sustainability. The involvement of young parents in the design and implementation is key to ensuring their dignity is upheld, their voices are heard and ownership of these interventions is secured.^59^ It is also critical for enhancing the cultural relevance, acceptability and sustainability of interventions, particularly in contexts where young parents face unique social and structural challenges. Future initiatives should therefore adopt more participatory and youth-centred approaches that position young parents not only as recipients but also as co-creators of interventions.

The majority of studies identified in this review reported interventions focusing on improving the social outcomes of young mothers.^47 48 50 52–57^ These studies generally reported positive effects of these interventions on young mothers, their caregivers and their children, including enhanced communication, decision-making, self-efficacy and parenting skills, alongside strengthened support from partners, parents and guardians. Such findings underscore the potential of social interventions to facilitate community reintegration, build self-confidence and foster positive parenting practices among young parents. Nonetheless, challenges were noted; in one study, the support extended to young mothers was interpreted by some community members as tacit approval of premarital sex and early pregnancy,^56^ pointing to the need for greater sensitivity to prevailing social norms when designing and implementing interventions.

Beyond social outcomes, two studies reported interventions that aimed to improve educational outcomes for young mothers,^49 56^ while two other studies addressed economic outcomes.^54 56^ Economic-related interventions typically provided vocational training, leadership and entrepreneurial skills to support income generation through employment or small business. These relatively few initiatives highlight an important gap, given the strong links between educational attainment, economic empowerment and long-term well-being for parenting adolescents and their children.^33^ Given evidence that structural determinants such as poverty,^60^ school dropout^61^ and exclusion from the labour market^62^ exacerbate the vulnerabilities of adolescent mothers and limit opportunities for themselves and their children, future interventions should adopt more holistic, multisectoral approaches that extend beyond immediate social and health needs to address structural determinants of young parents’ trajectories.

Our review highlights gaps in terms of interventions addressing gender-based violence, repeat pregnancy, school re-entry and contraceptive uptake among young parents. These areas represent critical dimensions of adolescent health and well-being, yet remain underexplored relative to mental health, parenting skills and general SRH knowledge. The lack of targeted programming to reduce gender-based violence is particularly concerning, given its strong association with adverse maternal and child health outcomes and its role in perpetuating cycles of vulnerability.^63^ Similarly, limited attention to repeat pregnancy, school re-entry and contraceptive uptake constrains opportunities for young mothers to regain control over their reproductive lives, pursue education and improve long-term socio-economic prospects.

A notable limitation of the existing evidence base is the limited use of rigorous evaluation designs. Only two studies employed a randomised controlled trial design,^45 46^ one of which was a pilot study,^45^ while most relied on study designs that do not support causal inference. This reliance on less robust methodologies constrains the ability to attribute observed outcomes directly to the interventions. It also limits generalisability and weakens the evidence needed to inform policy and scale-up. Strengthening the evidence base will require greater investment in rigorous designs, alongside mixed-methods approaches that capture both impact and contextual factors influencing effectiveness.

### Study strengths and limitations

The review adheres to the PRISMA-ScR guidelines, and it is one of the first scoping reviews to comprehensively map and analyse interventions aiming to improve young parents’ health and socio-economic well-being in Africa. However, it is not without limitations. One potential limitation is that we may have missed relevant studies/interventions that are unpublished, given that the review was limited to peer-reviewed articles or those published in languages other than English and French. Also, the substantial variation in intervention designs, populations and outcome measures precluded conducting a systematic review. In addition, we could not assess the certainty of the body of evidence for each outcome from this review given the multicomponent nature of interventions implemented contributing to multiple outcomes.

## Conclusion

The review points to existing interventions that have shown positive outcomes in addressing the health and socio-economic needs of young parents in Africa. However, the existing evidence base for interventions targeting African young parents’ well-being is limited in volume, geographical spread, evaluation rigour and focus on young fathers. The findings underscore the need for more robust research to identify effective interventions for this population.

## Supplementary material

10.1136/bmjopen-2026-117430 online supplemental file 1

10.1136/bmjopen-2026-117430 online supplemental file 2

10.1136/bmjopen-2026-117430 online supplemental file 3

10.1136/bmjopen-2026-117430 online supplemental file 4

10.1136/bmjopen-2026-117430 online supplemental file 5

## Data Availability

All data relevant to the study are included in the article or uploaded as supplementary information.
