# Supplementary material for: Interventions for improving the health and socio-economic well-being of young parents: a scoping review of studies in Africa
Source: BMJ Open. 2026 Sep 25;16(9):e117430. doi: 10.1136/bmjopen-2026-117430 (PMC13630077; doi:10.1136/bmjopen-2026-117430)
Supplement: Supplementary file 1 [file bmjopen-16-9-s001.pdf]

BMJ Open

Interventions for improving the health and socioeconomic well-being of young parents in Africa: a systematic review protocol

Sally Atieno Odunga, Beryl Machoka, Glorious Jesang, Grace Kathure Mugo, Winnan Lucia Munyasa, Sheila Mukabana, Beatrice W Maina, Anthony Idowu Ajayi, Caroline W Kabiru

To cite: Odunga SA, Machoka B, Jesang G, *et al*. Interventions for improving the health and socioeconomic well-being of young parents in Africa: a systematic review protocol. *BMJ Open* 2024;**14**:e083191. doi:10.1136/bmjopen-2023-083191

► Prepublication history and additional supplemental material for this paper are available online. To view these files, please visit the journal online (https://doi.org/10.1136/bmjopen-2023-083191).

Received 13 December 2023
Accepted 19 July 2024

Check for updates

© Author(s) (or their employer(s)) 2024. Re-use permitted under CC BY-NC. No commercial re-use. See rights and permissions. Published by BMJ.

Sexual, Reproductive, Maternal, Newborn, Child, and Adolescent Health (SRMNCAH) Unit, African Population and Health Research Center, Nairobi, Kenya

Correspondence to
Sally Atieno Odunga;
odungasally@gmail.com

ABSTRACT

Introduction Early childbearing is highly prevalent in Africa. Despite the harmful consequences of early childbearing on young people, there is limited documentation of interventions that aim to improve the health and socioeconomic well-being of young parents on the continent. In this systematic review, we will map and provide a critical synthesis of interventions that aim to improve the health and socioeconomic well-being of young parents in Africa to inform future policy and programmatic decision-making.

Methods and analysis The systematic review will adhere to the Preferred Reporting Items for Systematic Reviews and Meta-Analysis (PRISMA) guidelines. We will conduct an electronic search of peer-reviewed articles published in six databases: PubMed, Science Direct, AJOL, JSTOR, ERIC and Google Scholar. We will also search for grey literature through Google search and organisations' websites to broaden the number of interventions retrieved. Articles will be included if (1) the study participants are young mothers and fathers aged 10–24 years, (2) the article reports on interventions targeting young mothers and fathers in Africa or individual countries in Africa, (3) the article is published in English or French, (4) the article reports on health and socioeconomic well-being outcomes and (5) the article was published between 1 January 2000 and 31 December 2023. We will extract relevant information from articles that meet the inclusion criteria and synthesise data using both quantitative and qualitative approaches. Two reviewers will independently screen articles for inclusion, extract data from included articles and assess the methodological quality of studies.

Ethics and dissemination Ethical approval is not required for the systematic review since we are synthesising publicly available publications. Findings from this systematic review will be published in a peer-reviewed journal and further disseminated in conferences and convenings focusing on the health and socioeconomic well-being of young parents.

PROSPERO registration number CRD42023464828.

STRENGTHS AND LIMITATIONS OF THIS STUDY

⇒ This systematic review protocol follows the PRISMA (Preferred Reporting Items for Systematic Reviews and Meta-Analysis) guidelines and provides important insights to inform the main systematic review study.

⇒ To minimise the likelihood of reviewer bias, two reviewers will screen articles for eligibility and assess the methodological quality of included studies.

⇒ Given the scope, the review may fail to capture a significant number of interventions undertaken before 2000, those currently ongoing and those published in languages other than English and French.

INTRODUCTION

Early childbearing is one of the major threats to young people's[1 2] health and socioeconomic well-being (eg, ability to remain in school and access to financial resources), potentially hindering their smooth transition into adulthood.[3 4] Early childbearing has both immediate and lifelong consequences for young mothers and their children.[5 6] Young women are at a higher risk of maternal mortality than older women.[7 8] They are less likely to receive antenatal care, initiate antenatal care early or complete the recommended number of visits.[8–11] Furthermore, the incidence of stillbirths and infant mortality is 50% higher among babies born to mothers younger than 20 years compared with those born to mothers aged 20–29 years.[12] In addition to the heightened health risks, young mothers are more prone to social risks such as smoking and alcohol abuse,[13 14] which elevate the likelihood of stillbirth, low birth weight and other health issues for their babies.[15 16]

When young women and girls become pregnant, their education is jeopardised, impacting their employability and overall contribution to the economy.[17] For example, a report by the United Nations Population Fund (UNFPA) shows that if 200 000 young mothers and girls in Kenya were employed

BMJ Open: first published as 10.1136/bmjopen-2023-083191 on 5 August 2024. Downloaded from http://bmjopen.bmj.com/ on December 27, 2025 by guest. Protected by copyright, including for uses related to text and data mining, AI training, and similar technologies.

instead of having become pregnant, it would inject an additional $3.4 billion into the economy annually.[18] Besides health, educational and economic implications, pregnancy carries negative social repercussions for young women and girls. Young women and girls who become pregnant face stigmatisation, diminished social status within households and society, and early and forced marriage.[19] The consequences of early childbearing also extend beyond them and reverberate through families, societies and economies—affecting family relationships and dynamics as they adapt to the presence of a new family member.[20 21] Moreover, evidence by Ferraro *et al* indicates that early childbearing has intergenerational effects whereby children of young mothers are more likely to be young mothers themselves.[22]

Young fathers also face challenges that affect their health and socioeconomic well-being, although only little attention has been given to them.[23–26] For instance, they are sometimes forced to drop out of school to support their child financially.[24 26 27] Dropping out of school limits their employment opportunities and earnings, increasing the risks of persistent poverty levels and economic insecurity.[24 28] In addition, young fathers who are unable to provide for their children may experience diminished self-esteem[24] and may be denied access to their child by the child's mother and her family.[29 30] This in turn limits their involvement in the child's life.[26] Moreover, young fathers tend to experience greater psychological and emotional difficulties compared with their peers who are not fathers.[30] Despite these mental health difficulties, they have limited emotional and psychosocial support compared with young mothers.[26] Young fathers also face inadequate family support, with some experiencing hostility and punitive measures from their parents.[27 30] Similar to young mothers, early childbearing tends to be intergenerational among young fathers.[31]

Prioritising the well-being of young parents (mothers and fathers) will not only yield individual advantages but also contribute to far-reaching positive effects for broader societal outcomes, making it a strategic investment.[32] Existing research on young parents in Africa has predominantly focused on comprehending their experiences and pinpointing potential support interventions.[19 33 34] The few reviews of interventions targeting young parents have primarily centred on psychosocial interventions,[35–37] as well as interventions aimed at preventing repeat pregnancies.[38 39]

While these prior studies are important, no study, to the best of our knowledge, has systematically mapped interventions aimed at improving the health and socioeconomic well-being of young parents in the African context, where the burden of early childbearing is particularly high. Given the interconnectedness of cultural, social and economic factors that mould the experiences of young parents on the continent, we seek to systematically map and synthesise existing interventions aiming to improve their health and socioeconomic well-being. By doing so, we aim to generate insights to inform future programmes and policy interventions tailored to this subpopulation of young people.

METHODS

Study design

This protocol adheres to the Preferred Reporting Items for Systematic Review and Meta-analysis (PRISMA) standards.[40] The protocol has been registered with the International Prospective Register of Systematic Reviews (PROSPERO) at the University of York, UK. The registration number is CRD42023464828.

Search strategy

We will search six electronic databases—PubMed, Science Direct, AJOL, JSTOR, ERIC and Google Scholar—for published peer-reviewed articles. Additionally, we will review the reference lists of identified articles and review articles to identify additional articles. We will also search for grey literature through Google search and organisations' websites. The inclusion of grey literature is because there is a likelihood of finding very few studies since there is limited attention on research focusing on parenting adolescents. We will limit our search to articles reporting on studies conducted within the African region. To ensure that we capture the most recent evidence on interventions targeting young parents, only articles published between 1 January 2000 and 31 December 2023 will be included. Moreover, there is a growing focus on interventions to support young parents given the median age at first birth for women in many African countries is below the age of 20,[41 42] hence the need for recent evidence that can inform these programmes. References will be managed using Mendeley.

While the exact search terms will vary by database, we will structure our search around four key components/blocks: (1) interventions, (2) young parents (aged 10–24 years), (3) Africa and (4) health and socioeconomic well-being. These search terms will be adapted accordingly for every bibliographic database in combination with database-specific filters. We will employ Boolean operators ('AND', 'OR' and 'NOT') to maximise or narrow the specificity of the search. We will also use wildcards to search for any variations or alternate spellings of key concepts. We will place date, language (English and French) and age restrictions as part of our search criteria where applicable. The search strategies for the six databases are presented as an online supplemental document. The search will be guided by the PICOT (Population, Interventions, Comparison, Outcome and Time) framework as outlined in table 1.

A preliminary search to test the search strategy was conducted in August 2023 and will be updated once the final search is concluded to align with the timelines indicated in table 1.

Inclusion and exclusion criteria

We will include articles that meet the following criteria: (1) the article reports on a study where the participants

BMJ Open: first published as 10.1136/bmjopen-2023-083191 on 5 August 2024. Downloaded from http://bmjopen.bmj.com/ on December 27, 2025 by guest. Protected by copyright, including for uses related to text and data mining, AI training, and similar technologies.

Table 1 PICOT (Population, Interventions, Comparison, Outcome and Time) framework illustrating the scope of the systematic review

Framework item	Components
Population	Young parents aged 10–24 years in Africa
Interventions	Interventions/programmes/projects targeting or including young parents, such as peer-led interventions, young mothers' clubs, community-based interventions, support groups, parenting programmes
Comparison	We will not conduct any comparisons
Outcome	Health and socioeconomic well-being<br>► Health (mental health or psychosocial well-being, repeat pregnancy, sexual and reproductive health knowledge, transactional sex, lifestyle diseases, postnatal care, nutrition, family planning or contraception)<br>► Socioeconomic well-being (education, skill acquisition, financial literacy skills, parenting skills, socioeconomic empowerment)
Time	1 January 2000–31 December 2023

are young mothers and/or fathers aged 10–24 years or include a broader age range but present disaggregated results for young parents aged 10–24 years, (2) the article reports on interventions targeting young parents in Africa or individual countries in Africa, (3) the article is published in English or French, (4) the article reports on health and socioeconomic outcomes, and (5) the article was published between 1 January 2000 and 31 December 2023. We focus on young parents aged 10–24 years because programme implementers tend to broaden the age range when programming for young mothers (ie, they include girls older than 19 years). Further, WHO and UNFPA define young people as those aged 10–24 years[1 2] and so we considered anyone in that age range who has started childbearing as a young parent.

We will exclude articles if (1) the article reports on interventions that only target pregnant 10–24 year olds, (2) the article only reports on interventions targeting children of young parents, (3) the article reports on interventions aimed to improve pregnancy outcomes or (4) the full-text article is not accessible.

Study selection

A three-stage process will be used to identify articles that meet the inclusion criteria. First, we will remove all duplicate search results. Second, at least two independent reviewers will screen each article's title and abstract to assess its suitability for inclusion using Rayyan software. Lastly, two reviewers will independently conduct a full-text review of all articles selected in the second stage. Should any disagreements arise in the second and third stages, we will resolve them through a consensus discussion with all the authors. We will use the PRISMA flow chart diagram to illustrate the study article selection process as shown in figure 1

Data extraction

Data from all the included studies will be extracted in a data extraction form created using Microsoft Excel software. Two studies (quantitative and qualitative) selected for inclusion will be used to pilot test the form and modify it, if necessary. Two reviewers will extract data independently from the two studies. The reviewers will then compare the contents of the data extraction of the two studies for uniformity and consistency before proceeding to the final data extraction.

Two reviewers will independently extract data from each article and cross-check the information to enhance reliability and minimise errors. Reviewers will extract information about the articles including the title of the study, authors, publication year, subregion, country, sample size, age of participants, gender of participants, participants' marital status, study design, data type and study objectives. They will also extract information on the name and type of interventions and key findings on the effects of the intervention on the health and wellbeing of young parents in Africa. Discrepancies between reviewers will be resolved through team discussions and consensus.

Assessment of methodological quality of included articles

Two reviewers will independently assess the methodological quality of the included articles using the Critical Appraisal Skills Program (CASP) tool.[43] The CASP tool provides a systematic process through which the strengths and weaknesses of a research study can be identified.[43] The tool contains 10 key items, namely, (1) research aims, (2) research methodology, (3) research design, (4) recruitment, (5) data collection, (6) data analysis, (7) relationship between researcher and participants, (8) ethical considerations, (9) findings and (10) value of research. These 10 items are each scored using a three-point response scale (yes= '+', no= '–', can't tell='?'). Based on the assessment, the articles are finally assigned ratings of high, moderate and low quality. Any discrepancies in these rankings will be resolved through team discussion.

Data analysis and synthesis

After completing the data extraction process, we will process, organise and summarise the data. We will then synthesise data using both quantitative and qualitative approaches. To present the findings of this study, we will use the PRISMA framework. We anticipate recording data on heterogeneous interventions from a wide range of study designs, hence, we will group data based on the type of intervention. Within these groups, we will create subgroups based on the outcome, study design, participant characteristics and general study features.

BMJ Open: first published as 10.1136/bmjopen-2023-083191 on 5 August 2024. Downloaded from http://bmjopen.bmj.com/ on December 27, 2025 by guest. Protected by copyright, including for uses related to text and data mining, AI training, and similar technologies.

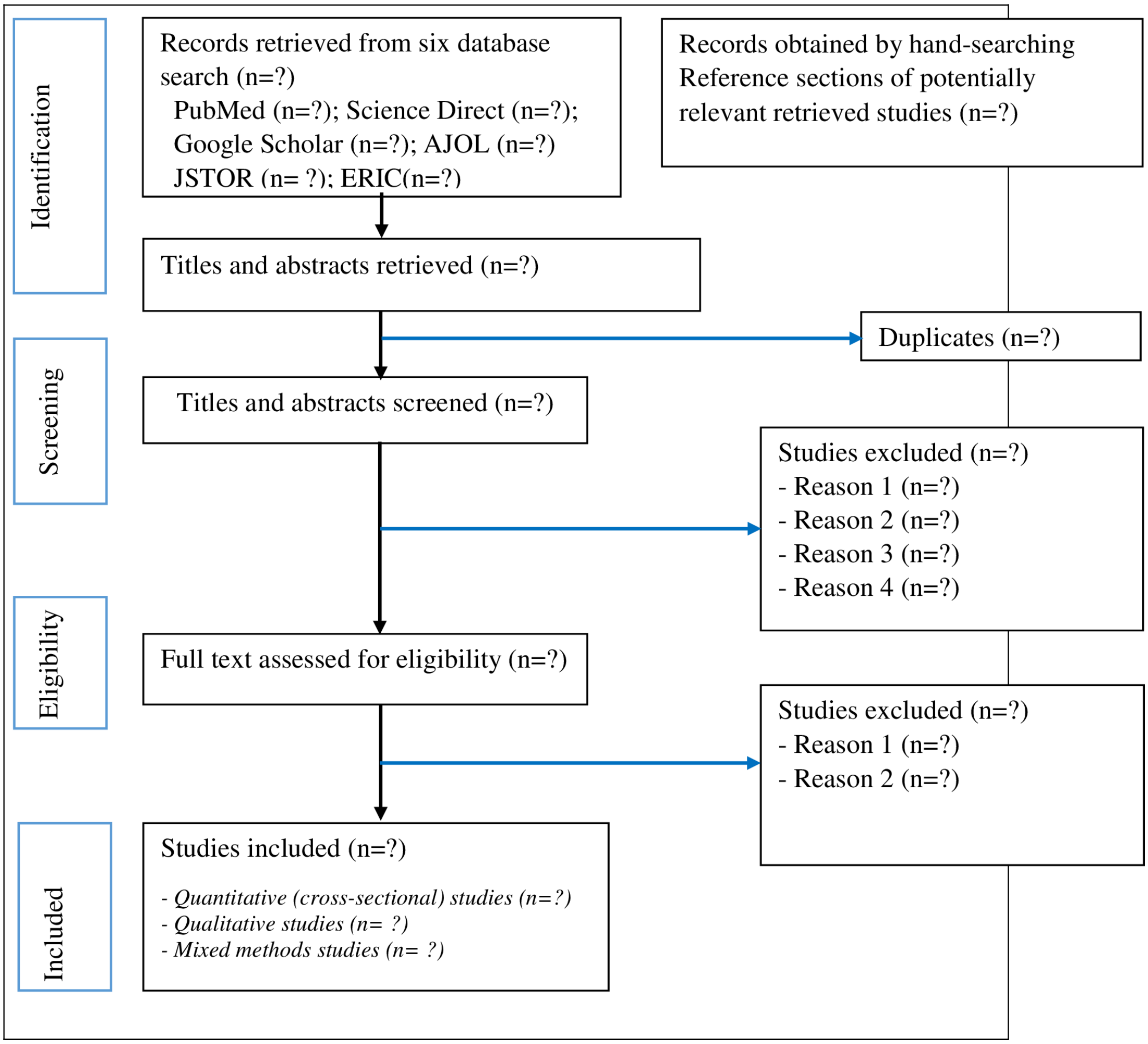

Figure 1 PRISMA (Preferred Reporting Items for Systematic Reviews and Meta-Analysis) flow diagram detailing the search and selection process.

Qualitative analysis

We will adopt a qualitative analysis approach given our objective is to describe the interventions for improving the health and socioeconomic well-being of young parents in Africa. We will use thematic analysis to identify key patterns and findings in the data extracted from qualitative articles. We will create coding categories based on the systematic review's objective and relevant data elements that inform it. To organise and process the data, we will use NVivo software for coding. During coding, the two reviewers will look for variations, differences or similarities in the results and classify data under the major subcategories. We will employ a narrative format to present findings on the interventions.

Patient and public involvement

We engaged stakeholders who have different expertise on young parents' health and socioeconomic well-being to develop the study design, research questions and search strategies. The stakeholders were researchers and non-researchers with specialisations in sexual and reproductive health, mental health, nutrition, youth empowerment and education. We will continue consulting them during the refining of search strategies, article retrieval, screening of articles, data extraction, data synthesis, data analysis, report writing, manuscript writing and dissemination of the findings. No patients will be involved in this study given the study design.

ETHICS AND DISSEMINATION

We do not require ethical approval for this systematic review since we are synthesising publicly available articles. We will submit the results of this systematic review for peer-reviewed publication and further disseminate the findings in conferences and other relevant fora.

Study strengths and limitations

This review is not without limitations. One potential limitation is that we may end up missing relevant studies/ interventions not published in peer-reviewed journals or that are unpublished, given that the review is limited to peer-reviewed publications. Second, reviewer bias could influence the papers included. To minimise the likelihood of reviewer bias, two reviewers will independently screen each article for study eligibility and assess the

BMJ Open: first published as 10.1136/bmjopen-2023-083191 on 5 August 2024. Downloaded from http://bmjopen.bmj.com/ on December 27, 2025 by guest. Protected by copyright, including for uses related to text and data mining, AI training, and similar technologies.

methodological quality of the included studies. These limitations notwithstanding, to our knowledge, this is one of the first systematic reviews focusing on interventions aimed at improving the health and socioeconomic well-being of young parents in Africa.

Contributors CWK, AIA, and BWM conceptualised and designed the study. SAO drafted the initial protocol with contributions to different sections from BM, GJ, GKM, SM and WLM. Coauthors developed the search strategy. All coauthors will contribute to the data extraction, analysis, interpretation and development of the manuscript. All authors critically reviewed the protocol before submission. The guarantor of the study is CWK who accepts full responsibility for the completed study, had access to the data and guided the decision to publish in consultation with the coauthors.

Funding This study was funded by a grant provided by the African Population and Health Research Center through its Strategic Innovations and Initiatives for a project titled 'Promoting holistic health and wellbeing of pregnant and parenting adolescents and their children in Africa—a life course model approach' (Grant No. 200.318). Staff costs were partially covered through funding to the African Population and Health Research Center from the Swedish International Development Cooperation Agency for the Challenging the Politics of Social Exclusion project (Sida Contribution No. 12103) and a grant from the International Development Research Centre (IDRC) for the Action to empower adolescent mothers in Burkina Faso and Malawi to improve their sexual and reproductive health project (Grant No. 109813-001). The views expressed in this article do not represent those of IDRC, its Board of Governors or SIDA.

Competing interests None declared.

Patient and public involvement Patients and/or the public were involved in the design, or conduct, or reporting or dissemination plans of this research. Refer to the Methods section for further details.

Patient consent for publication Not applicable.

Provenance and peer review Not commissioned; externally peer reviewed.

Supplemental material This content has been supplied by the author(s). It has not been vetted by BMJ Publishing Group Limited (BMJ) and may not have been peer-reviewed. Any opinions or recommendations discussed are solely those of the author(s) and are not endorsed by BMJ. BMJ disclaims all liability and responsibility arising from any reliance placed on the content. Where the content includes any translated material, BMJ does not warrant the accuracy and reliability of the translations (including but not limited to local regulations, clinical guidelines, terminology, drug names and drug dosages), and is not responsible for any error and/or omissions arising from translation and adaptation or otherwise.

Open access This is an open access article distributed in accordance with the Creative Commons Attribution Non Commercial (CC BY-NC 4.0) license, which permits others to distribute, remix, adapt, build upon this work non-commercially, and license their derivative works on different terms, provided the original work is properly cited, appropriate credit is given, any changes made indicated, and the use is non-commercial. See: http://creativecommons.org/licenses/by-nc/4.0/.

ORCID iDs
Sally Atieno Odunga http://orcid.org/0000-0002-0557-7971
Beatrice W Maina http://orcid.org/0000-0001-6205-3296

REFERENCES

1 The United Nations Population Fund. Adolescent and youth demographics: a brief overview.
2 World Health Organization. Adolescent health. Available: https://www.who.int/southeastasia/health-topics/adolescent-health [Accessed 17 Apr 2024].
3 Easterbrooks MA, Chaudhuri JH, Bartlett JD, *et al*. Resilience in parenting among young mothers: family and ecological risks and opportunities. *Child Youth Serv Rev* 2011;33:42–50.
4 Borkowski John G, Whitman Thomas L, Farris Jaelyn R. Adolescent mothers and their children: risks, resilience, and development. In: *Risk and resilience: adolescent mothers and their children grow up*. 2007: 1–34. Available: https://psycnet.apa.org/record/2007-07583-001
5 UNICEF DATA. Early childbearing and teenage pregnancy rates by country. Available: https://data.unicef.org/topic/child-health/adolescent-health/ [Accessed 24 Nov 2023].
6 Agnafors S, Bladh M, Svedin CG, *et al*. Mental health in young mothers, single mothers and their children. *BMC Psychiatry* 2019;19:112.
7 Nove A, Matthews Z, Neal S, *et al*. Maternal mortality in adolescents compared with women of other ages: evidence from 144 countries. *Lancet Glob Health* 2014;2:e155–64.
8 Lisonkova S, Potts J, Muraca GM, *et al*. Maternal age and severe maternal morbidity: a population-based retrospective cohort study. *PLoS Med* 2017;14:e1002307.
9 Simkhada B, Teijlingen ER van, Porter M, *et al*. Factors affecting the utilization of antenatal care in developing countries: systematic review of the literature. *J Adv Nurs* 2008;61:244–60.
10 Manyeh AK, Amu A, Williams J, *et al*. Factors associated with the timing of antenatal clinic attendance among first-time mothers in rural Southern Ghana. *BMC Pregnancy Childbirth* 2020;20:47.
11 Alem AZ, Yeshaw Y, Liyew AM, *et al*. Timely initiation of antenatal care and its associated factors among pregnant women in Sub-Saharan Africa: a multicountry analysis of demographic and health surveys. *PLoS One* 2022;17:e0262411.
12 World Health Organization. Early marriages, adolescent and young pregnancies. 2012. Available: https://apps.who.int/gb/ebwha/pdf_files/EB130/B130_12-en.pdf [Accessed 13 Dec 2023].
13 Chapman SLC, Wu L-T. Substance use among adolescent mothers: a review. *Child Youth Serv Rev* 2013;35:806–15.
14 Stone R. Pregnant women and substance use: fear, stigma, and barriers to care. *Health Justice* 2015;3:1.
15 Odendaal H, Dukes KA, Elliott AJ, *et al*. Association of prenatal exposure to maternal drinking and smoking with the risk of stillbirth. *JAMA Netw Open* 2021;4:e2121726.
16 Marufu TC, Ahankari A, Coleman T, *et al*. Maternal smoking and the risk of still birth: systematic review and meta-analysis. *BMC Public Health* 2015;15:239.
17 Bajracharya A, Psaki SR, Sadiq M. Child marriage, adolescent pregnancy and school dropout in South Asia. 2019.
18 United Nations Population Fund. Motherhood in childhood: facing the challenge of adolescent pregnancy.
19 Mangeli M, Rayyani M, Cheraghi MA, *et al*. Exploring the challenges of adolescent mothers from their life experiences in the transition to motherhood: a qualitative study. *J Family Reprod Health* 2017;11:165–73.
20 UNICEF. Early childbearing. 2022. Available: https://data.unicef.org/topic/child-health/adolescent-health/ [Accessed 24 Nov 2023].
21 Mangeli M, Rayyani M, Cheraghi MA. Factors that encourage early marriage and motherhood from the perspective of Iranian adolescent mothers: a qualitative study. *World Family Medicine Journal* 2017;15:67–74.
22 Ferraro AA, Cardoso VC, Barbosa AP, *et al*. Childbearing in adolescence: intergenerational dejà-vu? Evidence from a Brazilian birth cohort. *BMC Pregnancy Childbirth* 2013;13:1–8.
23 Osborn M. Young fathers: unseen but not invisible. *Fam Relatsh Soc* 2015;4:323–9.
24 Lau Clayton C. The lives of young fathers: a review of selected evidence. *Soc Policy Soc* 2016;15:129–40.
25 Holgate HS, Evans R, Yuen FKO. *Teenage pregnancy and parenthood*. 2006.
26 Neale B, Clayton CL. Following fathers: the lived experiences of teenage parenting overtime. 2011.
27 Makhavhu EM, Ramukumba TS, Masala-Chokwe ME. Exploring the dilemmas, challenges, and opportunities of adolescent fatherhood: an exploratory case study. *Inquiry* 2023;60:469580221146827.
28 Lemay CA, Cashman SB, Elfenbein DS, *et al*. A qualitative study of the meaning of fatherhood among young Urban fathers. *Public Health Nurs* 2010;27:221–31.
29 Reeves J, Gale L, Webb J, *et al*. Focusing on young men: developing integrated services for young fathers. *Comm Pract* 2009;82:18–21.
30 Bunting L, McAuley C. Research review: teenage pregnancy and parenthood: the role of fathers. *Child Fam Soc Work* 2004;9:295–303.
31 Sipsma H, Biello KB, Cole-Lewis H, *et al*. Like father, like son: the intergenerational cycle of adolescent fatherhood. *Am J Public Health* 2010;100:517–24.
32 Patton GC, Sawyer SM, Santelli JS, *et al*. Our future: a lancet commission on adolescent health and wellbeing. *Lancet* 2016;387:2423–78.
33 Ajayi AI, Athero S, Muga W, *et al*. Lived experiences of pregnant and parenting adolescents in Africa: a scoping review. *Reprod Health* 2023;20:113.
34 Muthengi E, Gitau T, Austrian K. Is working risky or protective for married adolescent girls in urban slums in kenya? Understanding the

BMJ Open: first published as 10.1136/bmjopen-2023-083191 on 5 August 2024. Downloaded from http://bmjopen.bmj.com/ on December 27, 2025 by guest. Protected by copyright, including for uses related to text and data mining, AI training, and similar technologies.

association between working status, savings and intimate-partner violence. *PLoS ONE* 2016;11:e0155988.
35 Laurenzi CA, Gordon S, Abrahams N, *et al*. Psychosocial interventions targeting mental health in pregnant adolescents and adolescent parents: a systematic review. *Reprod Health* 2020;17:65.
36 Lieberman K, Le HN, Perry DF. A systematic review of perinatal depression interventions for adolescent mothers. *J Adolesc* 2014;37:1227–35.
37 Sangsawang B, Wacharasin C, Sangsawang N. Interventions for the prevention of postpartum depression in adolescent mothers: a systematic review. *Arch Womens Ment Health* 2019;22:215–28.
38 DiCenso A, Guyatt G, Willan A, *et al*. Primary care among adolescents: systematic review of randomised controlled trials. *BMJ* 2002;324:1–9.
39 Frederiksen BN, Rivera MI, Ahrens KA, *et al*. Clinic-based programs to prevent repeat teen pregnancy: a systematic review. *Am J Prev Med* 2018;55:736–46.
40 Moher D, Shamseer L, Clarke M, *et al*. Preferred reporting items for systematic review and meta-analysis protocols (prisma-p) 2015 statement. *Syst Rev* 2015;4:1177–85.
41 Negash WD, Asmamaw DB. Time to first birth and its predictors among reproductive age women in high fertility countries in Sub-Saharan Africa: inverse weibull gamma shared frailty model. *BMC Pregnancy Childbirth* 2022;22:844.
42 Neal S, Channon AA, Chandra-Mouli V, *et al*. Trends in adolescent first births in Sub-Saharan Africa: a tale of increasing inequity? *Int J Equity Health* 2020;19:151.
43 CASP Checklists. Critical appraisal skills programme. Available: https://casp-uk.net/casp-tools-checklists/ [Accessed 25 Nov 2023].

BMJ Open: first published as 10.1136/bmjopen-2023-083191 on 5 August 2024. Downloaded from http://bmjopen.bmj.com/ on December 27, 2025 by guest . Protected by copyright, including for uses related to text and data mining, AI training, and similar technologies.
