# Supplementary material for: Interventions for improving the health and socio-economic well-being of young parents: a scoping review of studies in Africa
Source: BMJ Open. 2026 Sep 25;16(9):e117430. doi: 10.1136/bmjopen-2026-117430 (PMC13630077; doi:10.1136/bmjopen-2026-117430)
Supplement: Supplementary file 2 [file bmjopen-2026-117430.reviewer_comments.pdf]

PEER REVIEW HISTORY

BMJ Open publishes all reviews undertaken for accepted manuscripts. Reviewers are asked to complete a checklist review form and are provided with free text boxes to elaborate on their assessment. These free text comments are reproduced below.

ARTICLE DETAILS

Title (Provisional)

Interventions for improving the health and socioeconomic well-being of young parents: A scoping review of studies in Africa

Authors

Mugo, Grace Kathure; Jesang, Glorious; Odunga, Sally Atieno; Maina, Beatrice W.; Kabiru, Caroline W; Ajayi, Anthony Idowu

---

VERSION 1 - REVIEW

**Reviewer**	**1**
**Name**	**Moller, Ann-Beth**
**Affiliation**	**University of Gothenburg Institute of Medicine**
**Date**	**27-Mar-2026**
**COI**	**None**

Many thanks for the opportunity to review the manuscript “Interventions for improving the health and socioeconomic well-being of young parents: A scoping review of studies in Africa.” This is an important and timely topic, and the manuscript is generally well written. However, the methodological approach is not clearly presented. As currently described, it appears to mix elements of a systematic review with those of a scoping review, which creates confusion for the reader.

Below are specific comments for the authors to consider:

Title and Keywords:

- The title refers to “Africa,” but the manuscript focuses on “sub-Saharan Africa”. The title should reflect this geographical scope.

- The manuscript includes “two separate sets of keywords”:

1. Africa South of the Sahara, Health, Adolescent

2. Young parents, interventions, health and socioeconomic well-being, Africa

Please clarify why two lists are provided and consolidate them into one coherent set.

Methods

- It reads on p. 4. The substantial variation in intervention designs, populations, and outcome measures precluded conducting a systematic review. The authors conducted a scoping review and not a systematic review – please clarify.

- As this is a scoping review, the authors should follow the PRISMA-ScR reporting guidelines:

Tricco AC, Lillie E, Zarin W, et al. PRISMA Extension for Scoping Reviews (PRISMA-ScR): Checklist and Explanation. Ann Intern Med. 2018;169(7):467–73.

- On p. 7, the manuscript states that the review was limited to studies "conducted in the African region." Please define what is meant by "African region" and ensure consistent terminology throughout (e.g., SSA vs. African region).

- Scoping reviews typically use a PIO (Population–Intervention–Outcome) framework rather than PICOT. Please revise accordingly.

- Please specify which methodological framework guided the scoping review (e.g., Arksey & O'Malley, Levac et al., JBI or?).

- The inclusion of a risk of bias assessment is not aligned with scoping review methodology. According to JBI and other major frameworks, scoping reviews aim to map the evidence rather than assess study quality. Please justify or remove this component.

- The search strategy is insufficiently detailed. For transparency and reproducibility, please provide:

- the full search strategy for each database.

- the number of hits retrieved per database.

- Please also provide information on the data extraction process and share the extracted variables or data charting form.

Results

- Table 2: The title refers to "Target group and involvement of adolescent mothers in intervention design and implementation," but the table does not include information on the "implementer". Please revise the title or add the missing information.

- The tables should indicate where each study was conducted.

- Please add reference numbers to all studies listed in the tables, not only author names and publication years. The same applies to Tables 3 and 4.

- In Table 4, it is unclear why "Funders" are included. Please justify or remove this column.

- A list of abbreviations used in the manuscript is missing and should be added.

References

- Reference 7 (Chaaban & Cunningham, 2011) is quite dated. Please consider whether a more recent reference is available to support the argument.

Overall Assessment

The manuscript addresses an important topic and has the potential to make a valuable contribution. However, substantial revisions are required to ensure methodological clarity, adherence to scoping review standards, and improved transparency in reporting. I encourage the authors to revise the manuscript accordingly.

**Reviewer**	**2**
**Name**	**Fisher, Jane**
**Affiliation**	**Monash University, Jean Hailes Research Unit, School of Public Health and Preventative Medicine**
**Date**	**17-Apr-2026**
**COI**	**None**

Parenthood experienced by young people has potential adverse consequences for their lives and adult development and for the health and development of their children. In high-income countries where young people have access to post-secondary, and sexual and reproductive health education and to effective contraception and abortions, pregnancies among people under the age of 20 are now quite rare. However, in low- and middle-income countries, including many of the countries of Africa they remain common and are associated with intergenerational transfer of poverty.

This paper reports the findings of a scoping review of the evidence available about interventions to improve outcomes for young people in African countries who are experiencing pregnancy or parenthood. A protocol for the review had been published and it was registered with Prospero.

There are nevertheless aspects of this review that in my opinion require amendment to bring it to a publishable standard.

1. The protocol described the study as being a systematic review, but this has been submitted as a scoping review. Please explain this difference, why it was necessary and what the implications are?

2. There is no research question, please add one to the end of the Introduction.

3. Grey literature is not normally included in evidence syntheses because it reports work that has not been peer-reviewed. I think careful consideration should be given to this inclusion. If the grey literature is retained, then the separate search strategy used needs to be described in sufficient detail to enable replication. The findings about the grey literature are reported as being equivalent to those of the peer-reviewed studies and this is confusing, suggesting that they are of the same quality as the peer-reviewed literature. This needs to be addressed and corrected.

4. It is not clear why 24 papers were included in the review but methodological appraisal of only the 13 peer-reviewed studies was reported. This is a major flaw and the methodological quality of all the studies included in the review should be critically appraised and considered in the evidence synthesis. Consideration should be given to appraising them using the ACCODS checklist and thorough comparisons in the methodological quality of the two bodies of literature added.

5. It is not appropriate to cite global prevalence estimates of pregnancy rates among young people when there is enormous inter-country variation especially between high-income and LMICs. This variation should be provided.

6. The findings of the search and the PRISMA flow chart should be presented in the Results and not in the Methods

7. In the chart the peer-reviewed and grey literature should be distinguished.

8. The table reporting the findings does not include an account of what the comparator was – either usual care or another intervention, this needs to be added.

9. Although one column in Table 4 is headed 'Effects', no effects or effect sizes are provided. It is not possible to ascertain whether there was a difference at endline between groups and if so, what it was and, on the basis of what measure, and of what magnitude, and these need to be added.

10. It appears that only within group changes are documented, which are at best indicative evidence of the potential of the intervention, but not equivalent to a randomised controlled trial.

11. It is unclear what the study designs were or whether they were adequately powered to answer the research question. This information needs to be added.

12. Please use respectful person-first language wherever possible – young people who are parents rather than adolescent parents.

13. Please add a brief version of the search strategy to the abstract.

14. Please define 'adolescent childbearing' early in the paper and make it clear that it is a descriptor for both parents of only young women?

15. Please replace judgemental terms like 'gainfully employed' to more neutral terms like income -generating work.

16. The estimation of economic benefits appears only to be applied to the country and not to young women. Both should be included.

17. 'repeated early births' contribute to 'sustained cycles of early childbearing' using both descriptors appears redundant.

18. Page 9 line 10 'young parents in Africa, regardless of whether their children or pregnant adolescents were also targeted' does not make sense, please amend the language.

19. What are 'readmitted teenage mothers'? Please explain the term.

VERSION 1 - AUTHOR RESPONSE

Reviewer 1 Comments

Comment: Methodological approach is not clearly presented. As currently described, it appears to mix elements of a systematic review with those of a scoping review, which created confusion to the reader.

- **Response**: Thank you. We have revised the manuscript to clearly describe the study as a scoping review aimed at mapping available evidence on interventions targeting adolescent parents in Africa. The review has been adjusted to follow the JBI methodology for scoping reviews.

Comment: The title refers to "Africa" but the manuscript focuses on "sub-Saharan Africa". The title should reflect this geographical scope.

- **Response**: We reviewed the title and confirmed that the scope of the review is Africa, consistent with the terminology used throughout the manuscript. Therefore, the title has been retained. We have carefully reviewed the manuscript and ensured that this geographical scope is described consistently across all sections.

Comment: The manuscript includes "two separate sets of key words"; (i) Africa South of the Sahara, adolescent and; (ii) young parents, interventions, health and socioeconomic well-being, Africa. Please clarify why two lists are provided and consolidate them into one coherent list.

- **Response**: We used the most relevant key words available on the BMJ Open submission systems which are different from key words captured on our manuscript. We requested that key words available on the MeSH Database be included in the list of key words on the BMJ Open Submission System and list has been updated accordingly. Some of our key words, however, were not available on the MeSH Database and hence could not be included in the BMJ Open submission system.

Comment: It reads on page 4. The substantial variation in intervention designs, populations and outcome measures precluded conducting a systematic review. The authors conducted a scoping review and not a systematic review-please clarify.

- **Response**: The review was initially conceived as a systematic review, consistent with the registered protocol. However, during the review process, the evidence identified was found to be highly heterogeneous with respect to intervention types and outcome measures. Consequently, the review approach was revised to a scoping review, which was considered more appropriate for mapping and characterizing the available evidence.

Comment: As this is a scoping review, the authors should follow the PRISMA-ScR reporting guidelines: Tricco AC, Lillie E, Zarin W, et al. PRISMA Extension for Scoping Reviews (PRISMA-ScR): Checklist and Explanation. Ann Intern Med. 2018;169(7):467–73.

- **Response**: Thank you. The manuscript has been revised in accordance with the PRISMA-ScR reporting guidelines, and a PRISMA-ScR checklist filled in.

Comment: On p. 7, the manuscript states that the review was limited to studies "conducted in the African region." Please define what is meant by "African region" and ensure consistent terminology throughout (e.g., SSA vs. African region)

- **Response**: Terminology has been standardized throughout the manuscript. References to "African region and SSA" have been replaced with "Africa" where appropriate.

Comment: Scoping reviews typically use a PIO (Population–Intervention–Outcome) framework rather than PICOT. Please revise accordingly.

- **Response**: The review has been revised to follow the JBI scoping review using the PCC (Population-Concept-Context) framework.

Comment: The inclusion of a risk of bias assessment is not aligned with scoping review methodology. According to JBI and other major frameworks, scoping reviews aim to map the evidence rather than assess study quality. Please justify or remove this component.

- **Response**: The risk of bias assessment had been excluded since the JBI scoping review framework does not consider assessment of methodological quality of included studies as mandatory.

Comment: The search strategy is insufficiently detailed. For transparency and reproducibility, please provide: (i) the full search strategy for each data base and; the number of hits retrieved per database.

- **Response**: Detailed search strategy has been provided and search strategy for each data base and number of hits retrieved per data base attached as a supplementary material.

Comment: Please also provide information on the data extraction process and share the extracted variables or data charting form.

- **Response**: Additional information on the data extraction (charting) process has been added, including extracted variables, reviewer procedures, and the data-charting form, which is provided as supplementary material.

Comment: Table 2: The title refers to "Target group and involvement of adolescent mothers in intervention design and implementation," but the table does not include information on the "implementer". Please revise the title or add the missing information.

- **Response**: We agree that the previous title could be interpreted as implying the inclusion of information on intervention implementers. As the table focuses on the target groups and the involvement of young parents in intervention design or implementation, the title has been revised to more accurately reflect the information presented.

Comment: The tables should indicate where each study was conducted.

- **Response**: The countries where the studies were done have been included in every table.

Comment: Please add reference numbers to all studies listed in the tables, not only author names and publication years. The same applies to Tables 3 and 4.

- **Response**: Thank you. The reference numbers have been added in all the tables.

Comment: In Table 4, it is unclear why "Funders" are included. Please justify or remove this column.

- **Response**: We included the funders column because funding sources provide important contextual information, because as a scoping review aims to map the characteristics of the available evidence, reporting funders helps readers understand the institutional and financial landscape supporting these interventions and may inform future programme planning, implementation, and scale-up efforts. However, we have removed the funders section since it does not directly address our review question.

Comment: A list of abbreviations used in the manuscript is missing and should be added.

- **Response:** Thank you. A list of abbreviations has been added to the manuscript.

Comment: Reference 7 (Chaaban & Cunningham, 2011) is quite dated. Please consider whether a more recent reference is available to support the argument.

- **Response**: Thank you. A more recent reference has been added to support this statement, while retaining the original source where historically relevant.

i) <u>Reviewer two comments</u>

Comment: The protocol described the study as being a systematic review, but this has been submitted as a scoping review. Please explain this difference, why it was necessary and what the implications are?

- **Response**: The study changed to a scoping review due to diverse interventions and outcomes which couldn't be addressed using a systematic review methodology since systematic reviews focus on specific research question and intervention.

Comment: There is no research question, please add one to the end of the Introduction.

- **Response**: Thank you. A review question has been added at the end of the introduction section.

Comment: Grey literature is not normally included in evidence syntheses because it reports work that has not been peer-reviewed. I think careful consideration should be given to this inclusion. If the grey literature is retained, then the separate search strategy used needs to be described in sufficient detail to enable replication. The findings about the grey literature are reported as being equivalent to those of the peer-reviewed studies and this is confusing, suggesting that they are of the same quality as the peer-reviewed literature. This needs to be addressed and corrected.

- **Response**: Thank you. Since grey literature materials can't be equated to the peer-reviewed studies in terms of quality of evidence reported, we have decided to exclude use of grey literature from this study. We have attached summary tables showing the interventions that have been implemented among the young parents in Africa extracted from grey literature materials for learning purposes.

Comment: It is not clear why 24 papers were included in the review but methodological appraisal of only the 13 peer-reviewed studies was reported. This is a major flaw and the methodological quality of all the studies included in the review should be critically appraised and considered in the evidence synthesis. Consideration should be given to appraising them using the ACCODS checklist and thorough comparisons in the methodological quality of the two bodies of literature added.

- **Response**: Thank you. Grey literature materials have been excluded in this scoping review.

Comment: It is not appropriate to cite global prevalence estimates of pregnancy rates among young people when there is enormous inter-country variation especially between high-income and LMICs. This variation should be provided.

- **Response**: Thank you. A citation capturing the inter-country variations in adolescent child bearing rates has been included.

Comment: The findings of the search and the PRISMA flow chart should be presented in the Results and not in the Methods.

- **Response**: Thank you. The findings of the search and the PRISMA flow chart has been moved to the results section.

Comment: In the chart the peer-reviewed and grey literature should be distinguished.

- **Response**: The grey literature materials has been removed hence the separation of grey literature and peer reviewed studies in the flow diagram was not be necessary.

Comment: The table reporting the findings does not include an account of what the comparator was – either usual care or another intervention, this needs to be added.

- **Response**: Thank you. Table 2 has been included to capture the intervention and comparator details of the studies.

Comment: Although one column in Table 4 is headed 'Effects', no effects or effect sizes are provided. It is not possible to ascertain whether there was a difference at endline between groups and if so, what it was and, on the basis of what measure, and of what magnitude, and these need to be added.

- **Response**: Thank you. A detailed account of the effects and effect size between baseline and endline groups has been included in Table 5 where available.

Comment: It appears that only within group changes are documented, which are at best indicative evidence of the potential of the intervention, but not equivalent to a randomised controlled trial.

- **Response**: Thank you. Our review only captured two randomized controlled trials, one was a pilot study, and the findings were presented qualitatively showing the within group changes brought about by the intervention. Studies that used quasi-experimental methods, did show the differences in the effects of the intervention among the baseline and endline groups, or, the intervention and comparison groups. We agree that the findings of the review are best indicative of the potential of these interventions and that more rigorous designs need to be adopted to show the effectiveness of these interventions.

Comment: It is unclear what the study designs were or whether they were adequately powered to answer the research question. This information needs to be added.

- **Response**: Thank you. The study designs have been included in Table 1 in the revised manuscript.

Comment: Please use respectful person-first language wherever possible – young people who are parents rather than adolescent parents.

- **Response**: Thank you. The manuscript has been revised to consistently refer to them as young parents, or young fathers and young mothers.

Comment: Please add a brief version of the search strategy to the abstract.

- **Response**: Thank you. The abstract has been refined to adequately capture the search strategy.

Comment: Please define 'adolescent childbearing' early in the paper and make it clear that it is a descriptor for both parents of only young women?

- **Response**: Thank you. A definition has been provided in the narrative to make it clear that the term describes both parents and not young women only.

Comment: Please replace judgmental terms like 'gainfully employed' to more neutral terms like income -generating work

- **Response**: Thank you. We have replaced the term with income generating work or activity.

Comment: The estimation of economic benefits appears only to be applied to the country and not to young women. Both should be included.

- **Response**: Thank you. This has been addressed and citation provided.

Comment: 'repeated early births' contribute to 'sustained cycles of early childbearing' using both descriptors appears redundant.

- **Response**: Thank you. One descriptor has been removed.

Comment: Page 9 line 10 'young parents in Africa, regardless of whether their children or pregnant adolescents were also targeted' does not make sense, please amend the language.

- **Response**: Thank you. This has been amended accordingly.

Comment: What are 'readmitted teenage mothers'? Please explain the term.

- **Response**: Thank you. an alternative terminology has been used in the manuscript. These are teenage mothers who have been enrolled back to school after delivering their babies.

<u>Other comments received on 23rd June 2026</u>

Comment: During publication, our Production team will publish the author list using the information you provided in ScholarOne. The author list in your Title Page currently does not match the information listed in ScholarOne. We have attached the author list in ScholarOne (see page 1) and your Title Page. Please compare (the names, affiliations and departments) and confirm which author list should be used, and update the relevant sections.

- **Response**: Thank you. Both author lists have been aligned and they now match.

Comment: Reference citations should be cited in ascending order with Arabic numbering in either square brackets [1] or superscript. Please check your manuscript and ensure that all references formatted correctly. Please ensure that you do not use endnotes or footnotes. The references must not be linked between the intext citation and the reference list.

- **Response**: Thank you. The intext citations and the reference list are not linked and the reference citations have been formatted correctly using square brackets as instructed.

Comment: Your tables are too large to be included in the manuscript, specifically table 5. Please either reduce their size (maximum 2 pages / 9 columns) or upload them as supplementary material (please ensure you remove your original table and update the table citations within the manuscript).

- **Response**: Thank you. Table 5 has been reduced to two pages and table citations updated within the manuscript. The detailed summary of interventions capturing effects and effect sizes of the interventions in the included studies has been attached as supplementary file 5.

Comment: We note that your funders listed on the system and main document do not match. Please check and amend as needed noting that all information must be identical - including award IDS and grant numbers.

- **Response**: Thank you. Details of the funders have been aligned and updated accordingly.

VERSION 2 - REVIEW

**Reviewer**	**2**
**Name**	**Fisher, Jane**
**Affiliation**	**Monash University, Jean Hailes Research Unit, School of Public Health and Preventative Medicine**
**Date**	**07-Aug-2026**
**COI**	

The authors have made extensive revisions, and I think the paper is improved in terms of adherence to review and reporting methods and to precision and accuracy of language. The findings are discussed thoughtfully with useful indications about effective approaches to improve the health and social and economic circumstances of young people experiencing pregnancy in Africa.

There are nevertheless some amendments required:

• The aim is to describe 'what are some of the interventions…' whereas the study sought to identify and assess the quality of all the published trials and evaluations of interventions. The aim should be made more precise.

• Please explain why a systematic review 'could not be a correct approach'. Methodological diversity of the included studies is not a usual rationale for changing the design.

• Please remove the descriptor 'issue', which is too non-specific for academic research, from any use in the paper. Please replace it with a more precise term.

• It is still not clear why the account of prior knowledge was limited to sub-Saharan Africa when the title indicates that it applied to the whole of Africa. Please make this consistent throughout.

• Research stages (i) and (ii) (p 9 lines 51 – 53) are the same - this needs to be corrected. It is not clear why only five of the six JBI stages are followed – please explain and justify.

• What is the difference between 'key terms' and 'search terms'? Please explain the use of these descriptors.

• There is some inconsistency in the description of the exclusion criteria between the account in the abstract and summary and the account in the Methods. This is particularly in relation to whether or not they had been peer-reviewed. Please make this consistent throughout.

• Use of the term 'benefited' in the data table is confusing. It implies that there was a positive effect of the intervention, but this is unclear. For example the description of the Berry et al study 'the comparison group did not benefit from the parenting program' – it is not clear whether the group received it and it was not effective or did not receive it. Please make the descriptions accurate and precise.

VERSION 2 - AUTHOR RESPONSE

Authors response to reviewer 2 comments

Comment: The aim is to describe 'what are some of the interventions…' whereas the study sought to identify and assess the quality of all the published trials and evaluations of interventions. The aim should be made more precise.

- **Response**: Thank you. The aim has been amended and is now precise.

Comment: Please explain why a systematic review 'could not be a correct approach'. Methodological diversity of the included studies is not a usual rationale for changing the design.

- **Response**: Thank you. Since our review question was broad and sought to understand the existing body of evidence on interventions targeting parenting adolescent, a scoping review was a more appropriate approach that a systematic review, which seeks to answer a more precise question. We have explained this in the text

Comment: Please remove the descriptor 'issue', which is too non-specific for academic research, from any use in the paper. Please replace it with a more precise term.

- **Response**: Thank you. A more specific term for academic research has been used.

Comment: It is still not clear why the account of prior knowledge was limited to sub-Saharan Africa when the title indicates that it applied to the whole of Africa. Please make this consistent throughout.

- **Response**: Thank you. This has been amended accordingly and it is now consistent.

Comment: Research stages (i) and (ii) (p 9 lines 51 – 53) are the same - this needs to be corrected. It is not clear why only five of the six JBI stages are followed – please explain and justify.

- **Response**: Thank you. Amendments have been made and an explanation provided. Stage six of the JBI framework, which involves consulting stakeholders, is optional. However, we informally consulted with various colleagues working in relevant areas as the review progressed through the five steps.

Comment: What is the difference between ‘key terms’ and ‘search terms’? Please explain the use of these descriptors.

- **Response**: Thank you. One of the descriptors has been removed since they have the same meaning.

Comment: There is some inconsistency in the description of the exclusion criteria between the account in the abstract and summary and the account in the Methods. This is particularly in relation to whether or not they had been peer-reviewed. Please make this consistent throughout.

- **Response**: Thank you. The inconsistency in describing the exclusion criteria in the abstract and methods sections has been addressed.

Comment: Use of the term ‘benefited’ in the data table is confusing. It implies that there was a positive effect of the intervention, but this is unclear. For example, the description of the Berry et al study ‘the comparison group did not benefit from the parenting program’ – it is not clear whether the group received it and it was not effective or did not receive it. Please make the descriptions accurate and precise.

- **Response**: Thank you. The descriptions have been made more precise and clear for the reader.
