# Supplementary material for: Interventions for improving the health and socio-economic well-being of young parents: a scoping review of studies in Africa
Source: BMJ Open. 2026 Sep 25;16(9):e117430. doi: 10.1136/bmjopen-2026-117430 (PMC13630077; doi:10.1136/bmjopen-2026-117430)
Supplement: Supplementary file 3 [file bmjopen-16-9-s002.docx]

**Interventions for improving the health and socioeconomic well-being of young parents: A scoping review of studies in Africa.**

**Detailed search strategy across all data bases**

**PubMed search - 111 results**

**((((((((((((((((((((((((((Interventions) OR (programs)) OR (programmes)) OR (projects)) OR (initiatives)) OR ("adolescent mothers club")) OR ("peer support")) OR ("vocational training")) OR (evaluation)) OR (support)) OR ("life skill")) OR (training)) OR (psychosocial)) OR (prevent)) OR (promote)) NOT (USA)) NOT ("United Kingdom")) NOT (China)) NOT (Australia)) NOT (Europe)) NOT (Canada)) NOT ("United States")) NOT (America)) NOT ("United States of America")) AND ((((((((((((((((((((((((("parenting adolescents") OR ("adolescent mothers")) OR ("adolescent fathers")) OR ("teenage mother")) OR ("teenage father")) OR ("teen mom")) OR ("young mom")) OR ("young mother")) OR ("young parents")) OR ("adolescent parents")) OR ("juvenile mother")) OR ("teenage parents")) OR ("juvenile parents")) OR ("adolescent mom")) OR ("juvenile mom")) OR ("teen mother")) NOT (USA)) NOT ("United Kingdom")) NOT (China)) NOT (Australia)) NOT (Europe)) NOT (Canada)) NOT ("United States")) NOT (America)) NOT ("United States of America"))) AND ((((((((((((((((((((((((((((((((((((((((((((((((((((((((((((Africa) OR ("Sub-Saharan Africa")) OR (Algeria)) OR (Angola)) OR (Benin)) OR (Botswana)) OR ("Burkina Faso")) OR (Burundi)) OR ("Cabo Verde")) OR ("Cape Verde")) OR (Cameroon)) OR ("Central African Republic")) OR (Chad)) OR (Comoros)) OR ("Côte d'Ivoire")) OR ("Ivory Coast")) OR ("Democratic Republic of Congo")) OR (Djibouti)) OR (Egypt)) OR ("Equatorial Guinea")) OR (Eritrea)) OR (Eswatini)) OR (Swaziland)) OR (Ethiopia)) OR (Gabon)) OR (Gambia)) OR (Ghana)) OR (Guinea)) OR ("Guinea-Bissau")) OR (Kenya)) OR (Liberia)) OR (Lesotho)) OR (Libya)) OR (Madagascar)) OR (Malawi)) OR (Mali)) OR (Mauritania)) OR (Mauritius)) OR (Morocco)) OR (Mozambique)) OR (Namibia)) OR (Niger)) OR (Nigeria)) OR ("Republic of Congo")) OR (Rwanda)) OR ("Sao Tome and Principe")) OR (Senegal)) OR (Seychelles)) OR ("Sierra Leone")) OR (Somalia)) OR ("South Africa")) OR ("South Sudan")) OR (Sudan)) OR (Tanzania)) OR (Togo)) OR (Tunisia)) OR (Uganda)) OR (Zambia)) OR (Zimbabwe)) NOT (America))) AND (((((((((((((((((((((((((((((((((((("Health and wellbeing") OR (education)) OR ("school re-entry")) OR ("school retention")) OR ("school completion")) OR ("school transition")) OR ("mental health")) OR (depression)) OR ("psychosocial wellbeing")) OR ("Sexual Reproductive Health Knowledge")) OR ("transactional sex")) OR ("contraceptive uptake")) OR ("contraceptive use")) OR ("repeat pregnancy")) OR ("lifestyle choices")) OR ("financial literacy")) OR ("skill acquisition")) OR (employment)) OR ("parenting skills")) OR ("socioeconomic empowerment")) OR ("substance abuse")) OR ("school attendance")) OR ("school enrolment")) OR ("postnatal care")) OR (Nutrition)) OR (lactating)) OR (Breastfeeding)) NOT (USA)) NOT ("United Kingdom")) NOT (China)) NOT (Australia)) NOT (Europe)) NOT (Canada)) NOT ("United States")) NOT (America)) NOT ("United States of America"))** Filters: **English, from 2000/1/1 - 2024/12/31**

**2.	Science Direct-489 articles**

*Focused on changing the outcome only per search given the limited Boolean operators/characters one can use in a single search. We have searched all the alternative outcomes The first three terms are comprehensive enough to get relevant papers and narrow the search*

**1st combination-Health and wellbeing -25 articles**

(Interventions OR Programs) AND ("parenting adolescents" OR "adolescent mothers") AND (Africa OR "Sub-Saharan Africa") AND ("health and wellbeing") AND NOT America

**2nd combination-Education-144 articles**

**(**Interventions OR Programs) AND ("parenting adolescents" OR "adolescent mothers") AND (Africa OR "Sub-Saharan Africa") AND (education) AND NOT America

**3rd	combination-School re-entry-2 articles**

(Interventions OR Programs) AND ("parenting adolescents" OR "adolescent mothers") AND (Africa OR "Sub-Saharan Africa") AND ("school re-entry ") AND NOT America

**4th	combination-School retention-3 articles**

(Interventions OR Programs) AND ("parenting adolescents" OR "adolescent mothers") AND (Africa OR "Sub-Saharan Africa") AND ("school retention") AND NOT America

**5th	combination-School completion-1 article**

(Interventions OR Programs) AND ("parenting adolescents" OR "adolescent mothers") AND (Africa OR "Sub-Saharan Africa") AND ("school completion") AND NOT America

**6th combination-Mental Health-72 articles**

(Interventions OR Programs) AND ("parenting adolescents" OR "adolescent mothers") AND (Africa OR "Sub-Saharan Africa") AND ("mental health") AND NOT America

**7th combination-Depression-62 articles**

(Interventions OR Programs) AND ("parenting adolescents" OR "adolescent mothers") AND (Africa OR "Sub-Saharan Africa") AND (depression) AND NOT America

**8th combination-Psychosocial wellbeing- 4 articles**

(Interventions OR Programs) AND ("parenting adolescents" OR "adolescent mothers") AND (Africa OR "Sub-Saharan Africa") AND ("psychosocial wellbeing") AND NOT America

**9th combination-Sexual Reproductive Health knowledge -1 article**

(Interventions OR Programs) AND ("parenting adolescents" OR "adolescent mothers") AND (Africa OR "Sub-Saharan Africa") AND ("Sexual Reproductive Health knowledge") AND NOT America

**10th combination –Transactional sex-8 articles**

(Interventions OR Programs) AND ("parenting adolescents" OR "adolescent mothers") AND (Africa OR "Sub-Saharan Africa") AND ("transactional sex") AND NOT America

**11th combination-contraceptive uptake- 2 articles**

(Interventions OR Programs) AND ("parenting adolescents" OR "adolescent mothers") AND (Africa OR "Sub-Saharan Africa") AND ("contraceptive uptake") AND NOT America

**12th combination-contraceptive use- 47 articles**

(Interventions OR Programs) AND ("parenting adolescents" OR "adolescent mothers") AND (Africa OR "Sub-Saharan Africa") AND ("contraceptive use") AND NOT America

**13th combination-repeat pregnancy- 9 articles**

(Interventions OR Programs) AND ("parenting adolescents" OR "adolescent mothers") AND (Africa OR "Sub-Saharan Africa") AND ("repeat pregnancy") AND NOT America

**14th combination-financial literacy-2 articles**

(Interventions OR Programs) AND ("parenting adolescents" OR "adolescent mothers") AND (Africa OR "Sub-Saharan Africa") AND ("financial literacy") AND NOT America

**15th combination-skill acquisition-8 articles**

(Interventions OR Programs) AND ("parenting adolescents" OR "adolescent mothers") AND (Africa OR "Sub-Saharan Africa") AND ("skill acquisition") AND NOT America

**16th combination-employment-52 articles**

(Interventions OR Programs) AND ("parenting adolescents" OR "adolescent mothers") AND (Africa OR "Sub-Saharan Africa") AND (employment) AND NOT America

**17th combination-parenting skills-15 articles**

(Interventions OR Programs) AND ("parenting adolescents" OR "adolescent mothers") AND (Africa OR "Sub-Saharan Africa") AND ("parenting skills") AND NOT America

**18th combination-socioeconomic empowerment-1 article**

(Interventions OR Programs) AND ("parenting adolescents" OR "adolescent mothers") AND (Africa OR "Sub-Saharan Africa") AND ("socioeconomic empowerment") AND NOT America

**19th combination-substance use-41 results**

(Interventions OR Programs) AND ("parenting adolescents" OR "adolescent mothers") AND (Africa OR "Sub-Saharan Africa") AND ("substance use") AND NOT America

**20th combination-school attendance-16 articles**

(Interventions OR Programs) AND ("parenting adolescents" OR "adolescent mothers") AND (Africa OR "Sub-Saharan Africa") AND ("school attendance") AND NOT America

**21st combination-school enrolment-10 articles**

(Interventions OR Programs) AND ("parenting adolescents" OR "adolescent mothers") AND (Africa OR "Sub-Saharan Africa") AND ("school enrolment") AND NOT America

**3.	JSTOR-103 articles**

*Same case with Science direct in terms of limitation of using a particular number of search terms in a single search. We have used key alternatives of the outcome of health and wellbeing i.e. health and wellbeing, education, mental health and repeat pregnancy.*

**1st combination-Health and wellbeing -1 article**

(((((((Interventions) OR (Programs)) AND ("parenting adolescents")) OR ("adolescent mothers")) AND (Africa)) AND ("health and wellbeing")) NOT (America)) AND la:(en OR en)

**2nd combination- education-76 articles**

(((((((Interventions) OR (Programs)) AND ("parenting adolescents")) OR ("adolescent mothers")) AND (Africa)) AND (education)) NOT (America)) AND la:(eng OR en)

**3rd combination-mental health-22 articles**

(((((((Interventions) OR (Programs)) AND ("parenting adolescents")) OR ("adolescent mothers")) AND (Africa)) AND ("mental health")) NOT (America)) AND la:(eng OR en)

**4th combination- repeat pregnancy-3 articles**

(((((((Interventions) OR (Programs)) AND ("parenting adolescents")) OR ("adolescent mothers")) AND (Africa)) AND ("repeat pregnancy")) NOT (America)) AND la:(eng OR en)

**4.	Google scholar-239 articles**

*Same case with Science direct and JSTOR in terms of limitation of using a particular number of search terms in a single search. Focused on alternatives of key outcome: health and wellbeing, education, mental health*

**Combination-Health and wellbeing, education and mental health \**

(Interventions OR Programs) AND ("parenting adolescents" OR "adolescent mothers") AND (Africa OR "Sub-Saharan Africa") AND ("health and wellbeing" OR education OR “mental health”) -America-"United Kingdom"- Europe -Australia –China-Canada

**5.	ERIC- 128 articles**

*Focused on key search terms i.e. Interventions and adolescent mothers and adolescent fathers. This is because if other search terms such as health and wellbeing and Africa is applied, no paper is retrieved. Excluded countries below (helped narrow the search to Africa) and applied time filter*

Interventions AND "adolescent mothers" OR "adolescent fathers" -America-Europe-"United Kingdom"-Australia-China pubyearmin2000

**6.	AJOL-287 articles**

(Interventions OR Programs OR Programmes OR Projects OR Initiatives OR "Adolescent mothers club" OR "Peer support" OR “Vocational training "OR Evaluation OR Support OR "Life skill" OR Training OR Psychosocial OR Prevent OR Promote) AND ("parenting adolescents" OR "adolescent mothers" OR "adolescent fathers" OR "teenage mother" OR "teenage father" OR "teen mom" OR "young mom" OR "young mother" OR "young parents" OR "adolescent parents" OR "juvenile mother" OR "teenage parents" OR "juvenile parents" OR "adolescent mom" OR "juvenile mom" OR "teen mother") AND (Africa OR “Sub-Saharan Africa” OR Algeria OR Angola OR Benin OR Botswana OR "Burkina Faso" OR Burundi OR "Cabo Verde" OR "Cape Verde" OR Cameroon OR "Central African Republic" OR Chad OR Comoros OR "Côte d'Ivoire" OR "Ivory Coast" OR "Democratic Republic of Congo" OR Djibouti OR Egypt OR "Equatorial Guinea" OR Eritrea OR Eswatini OR Swaziland OR Ethiopia OR Gabon OR Gambia OR Ghana OR Guinea OR "Guinea-Bissau" OR Kenya OR Lesotho OR Liberia OR Libya OR Madagascar OR Malawi OR Mali OR Mauritania OR Mauritius OR Morocco OR Mozambique OR Namibia OR Niger OR Nigeria OR "Republic of Congo" OR Rwanda OR "Sao Tome and Principe" OR Senegal OR Seychelles OR "Sierra Leone" OR Somalia OR "South Africa" OR "South Sudan" OR Sudan OR Tanzania OR Togo OR Tunisia OR Uganda OR Zambia OR Zimbabwe) AND ("health and wellbeing" OR education OR "school re-entry" OR "school retention" OR "school completion" OR "school transition" OR "mental health" OR depression OR "psychosocial wellbeing" OR "Sexual Reproductive Health knowledge" OR "transactional sex" OR "contraceptive uptake" OR "contraceptive use" OR "repeat pregnancy" OR "Lifestyle choices" OR "financial literacy" OR "skill acquisition" OR employment OR "parenting skills" OR "socioeconomic empowerment" OR "substance use" OR "school attendance" OR "school enrolment") -USA-"United Kingdom"-China-Australia-Europe-Canada-"United States"-America-"United States of America"
