# Supplementary material for: Interventions for improving the health and socio-economic well-being of young parents: a scoping review of studies in Africa
Source: BMJ Open. 2026 Sep 25;16(9):e117430. doi: 10.1136/bmjopen-2026-117430 (PMC13630077; doi:10.1136/bmjopen-2026-117430)
Supplement: Supplementary file 4 [file bmjopen-16-9-s004.docx]

**Summary of interventions aimed at improving health and socioeconomic well-being of young parents in Africa.**

| **Author (Year)/ Implementer** | **Country** | **Description of intervention** | **Outcome (s)** | | **Effects** |  |
| --- | --- | --- | --- | --- | --- | --- |
|  |  |  | **Health** | **Social, economic and educational outcomes** |  | **Funders** |
| **Grey Literature** | | | | | | |
| Advocacy for Social Inclusion and Girls Education (ASIGE) (2022) | Ghana | Basketry development, vocational training and reproductive health education for teenage mothers. | Not indicated | Not indicated | Not provided | Americans Serving Abroad |
| Africaid and Ministry of Health and Child Care (2018) | Zimbabwe | Young mentor mothers (YMMs) intervention, where YMMs support their peers by conducting home visits and sending SMS reminders as well as through facility and community-based groups. | ART adherence  Viral load suppression | Not indicated | Improvement in viral suppression observed | Swedish International Development Cooperation Agency (Sida) |
| African Population and Health Research Center (APHRC) (2022) | Kenya | Sasa Mama Teen toolkit to improve the skills, confidence and coping mechanisms of pregnant and postpartum adolescent girls. | Not provided | Not provided | Not provided | Bill and Melinda Gates Foundation - Grand Challenges Explorations Initiative |
| Afya Uzazi (2020) | Kenya | Binti Shujaa (My Sister’s heroine”) Intervention is an innovative initiative used to address the health needs of adolescent mothers, pregnant adolescents and their children younger than 23 months old. The model used a peer-based and mentorship approach to link the target group with health and socio-economic services. The project was implemented through school-based and community-based sessions | Comprehensive sexual education, modern contraceptives, antenatal services, and HIV counseling and testing to young mothers | Empowering young mothers to make informed decisions on their sexual and reproductive health | Reduces unintended pregnancies among young mothers by improving the modern contraceptives uptake | USAID East Africa |
| Forum for African Women Educationists, Kenya and ENAI Africa (2023) | Kenya | FAWE model schools run Tuseme clubs for information sharing and mentoring to empower students. Teenage mothers receive capital funds and training in business management and entrepreneurial skills to support their small-scale businesses | Not indicated | Reentry into school and empowering them with entrepreneurial skills. | Improvement in school reentry and establishment of business and enhanced skills. | Ford Foundation |
| Impact Direct (2023) | Ghana | Skills training for teenage mothers | Not indicated | Professional, entrepreneurial and leadership skills. Income generation and business ownership | Improvement in professional, entrepreneurial, and leadership skills. | Not indicated on the webpage |
| International Centre for Reproductive Health Kenya (ICRHK), UNFPA, County Government of Kilifi (2019) | Kenya | Community health volunteers sensitized pregnant adolescents and young mothers on the services provided at the First Time Young Mothers Clinic. | Comprehensive sexual and reproductive health, maternal, and child health | Personal agency and community champions | Improved teenage mothers’ access to essential information and services related to their sexual and reproductive health. mothers.  Enhanced capacities of young mothers to make informed choices about family planning methods, birth planning and spacing. | UNFPA |
| Pediatric Adolescent Treatment Africa (PATA) (2018) | Malawi  Zambia  Tanzania  Uganda | Ask-Boost-Connect-Discuss (ABCD) was co-created with young mothers living with HIV in 2018 to provide group-based psychosocial support delivered by their trained peers | Better peer relationships Awareness of mental health and healthcare access | Positive thinking and confidence | Improved psychosocial well-being - Better adherence and retention in **Antiretroviral Therapy** care | Bill and Melinda Gates Foundation, Elma Foundation, Fogarty International Centre, International AIDS Society Cipher Fellowship, Oak Foundation and OFIL, UK Research and Innovation, GCRF Accelerate Hub, Center for AIDS Research at Emory University |
| Trocaire and Dutimbere ONG (2022) | Rwanda | Women’s economic empowerment through technical and vocational education and training provided to teenage mothers. | Not indicated | Socio- economic empowerment | Creation of new income-generating opportunities, Enhanced savings ability, reduced stigma and social exclusion, Increased self-confidence and esteem among the teen mothers. | Private Donor |
| UN Women, Imbuto Foundation and National Child Development agency (2021) | Rwanda | Psychosocial support counselling, parent-adolescent communication sessions, community-based outreach campaigns to increase awareness of gender-based violence and its effects, male involvement in fighting social discrimination, stigma and violence as well as promotion of income generating activities | Psychological well-being | Income generation and social wellbeing | Increased sense of dignity, self-worth, and  income-generating activities | Swedish Development Cooperation |

# References

1.	African Population and Health Research Center (APHRC). WHAT DO I DO NOW? A toolkit for helping adolescent mothers manage stress.

2.	Africaid M of H and CC. Young Mentor Mothers Initiative: A peer-support model tailored for young mothers and their infants in Zimbabwe | Children & AIDS [Internet]. [cited 2024 Aug 15]. Available from: https://www.childrenandaids.org/node/1034

3.	IMPACT direct. Skills training for Teen Mums – iMPACT direct [Internet]. [cited 2024 Aug 15]. Available from: https://impactdirect.eu/pinasol-foundation-and-impact-direct-foundation-launches-teen-mums-project-is-ghana/

4.	UN Women IFNCDA. UN Women provides psychosocial support to teen mothers in Rwanda | UN Women – Africa [Internet]. [cited 2024 Aug 15]. Available from: https://africa.unwomen.org/en/news-and-events/stories/2021/08/un-women-and-partners-in-rwanda-provide-access-to-srhr-information-and-awareness-on-gbv

5.	Pediatric Adolescent Treatment Africa (PATA). Ask-Boost-Connect-Discuss (ABCD) Programme - PATA [Internet]. [cited 2024 Aug 15]. Available from: https://teampata.org/programmes/ask-boost-connect-discuss-abcd/

6.	International Centre for Reproductive Health Kenya (ICRH). First Time Young Mothers (Phase 1) - International Centre for Reproductive Health (ICRH)-Kenya [Internet]. [cited 2024 Aug 15]. Available from: https://www.icrhk.org/first-time-young-mothers-project/

7.	Forum for African Women Educationalists (FAWE). Tamatisha project | Forum for African Women Educationalists Kenya Chapter [Internet]. [cited 2024 Aug 15]. Available from: https://fawe.or.ke/tamatisha-project/

8.	Trocaire DO. Vocational training and savings groups changing the lives of teen mothers in Rwanda - Trócaire [Internet]. [cited 2024 Aug 15]. Available from: https://www.trocaire.org/news/vocational-training-and-savings/

9.	Advocacy for Social Inclusion and Girls Education (ASIGE). ADVOCACY FOR SOCIAL INCLUSION AND GIRLS EDUCATION (ASIGE) – Catalyst 2030 [Internet]. [cited 2024 Aug 15]. Available from: https://catalyst2030.net/organisations/advocacy-for-social-inclusion-and-girls-education-asige/

10.	Afya Uzazi. More mothers embrace exclusive breastfeeding | by Afya Uzazi Nakuru-Baringo Program | Medium [Internet]. [cited 2024 Aug 15]. Available from: https://afyauzazi.medium.com/more-mothers-embrace-breastfeeding-e191acace5
