# Supplementary material for: Interventions for improving the health and socio-economic well-being of young parents: a scoping review of studies in Africa
Source: BMJ Open. 2026 Sep 25;16(9):e117430. doi: 10.1136/bmjopen-2026-117430 (PMC13630077; doi:10.1136/bmjopen-2026-117430)
Supplement: Supplementary file 5 [file bmjopen-16-9-s005.docx]

**Interventions for improving the health and socioeconomic well-being of young parents: A scoping review of studies in Africa**

**Table 5: Detailed summary of interventions targeting young parents in Africa.**

| **Author (Year)/ Implementer** | **Country** | **Description of intervention** | **Outcome (s)** | | **Effects and effect sizes of the interventions** |
| --- | --- | --- | --- | --- | --- |
|  |  |  | **Health** | **Social, economic and educational outcomes** |  |
| Berry et al (2022) | South Africa | Adolescent parenting program adopted a group-based approach to break the cycle of teenage pregnancy and prevent child abuse in Cape Town, South Africa. The intervention had two focus areas, namely developing positive parenting skills and supporting adolescent development for responsible adulthood. The study further assessed whether depression influences parenting outcomes. | Depression | Parenting skills and resilience | The intervention and control groups improved in positive parenting (intervention: paired t-test=-4.50, p<0.001; Control: t-test =-3.27; p=0.002) with no statistically significant inter-group difference.  There were no changes in the percentage of biological adolescent parents with high depression risk. Individual resilience increased at post-intervention (p=0.008) and follow-up (p=0.034) assessments. |
| Chingono et al (2022) | Zimbabwe | Self-help groups led by a trained facilitator on problem-solving skills, practice-based learning for healthy parenting and safer sexual practices, the importance of self-care and appropriate health- seeking, and improving capacity to manage and save money | Mental well-being | Self-confidence  Saving and investing money | Self-help groups were seen as a source of hope for adolescent mothers. Participants joined groups to obtain support and reported that their mental well-being improved due to new social networks, feelings of solidarity with peers, and increased confidence for positive action, that is, seeking health services for themselves and their babies.  Prior to enrolment, 16% showed signs of possible common mental disorders falling to 2% at follow-up. This contributed to enhancement of their mental health outcomes as well as their social outcomes due to improved self-confidence. |
| Gage et al (2024) | DRC | Momentum Project interventions offered an integrated family planning and maternal and child health as well as nutrition activities targeting married and unmarried first-time mothers (FTMs) aged 15-24 years and their male partners in Kinshasa, Democratic Republic of Congo. | Contraceptive uptake  Mother and child health  Nutrition | Contraceptive use norms, behavior and personal agency | The intervention had greater effects on postpartum family planning behaviour than on postpartum norms (more positive behavioural effects than normative effects) and no effect on normative change among never married 15–19-year-olds.  There was a 22-percentage increase in the probability of FTM perceiving that significant others expected her to discuss postpartum FP with her male partners before childbirth compared to the comparison group among never married FTMs age 20-24 years. |
| Gureje et al (2022) | Nigeria | Behavioral activation and problem-solving treatment (PST), parenting skills training, and social and parenting skills support provided by a self-identified adult “neighborhood mother” | Depressive symptoms | Parenting skills | Evaluation was conducted to establish the effectiveness of a psychosocial intervention where by the intervention group had lower levels of depressive symptoms than the control group, mean EPDS scores: 5∙5 (SD-3∙6) versus 7∙2 (SD-4∙0) (adjusted mean difference -1∙84 (95%CI- 3∙06 to 􀀀 0∙62; p = 0∙003) and better parenting practices, mean total HOME-IT scores: 29∙8 (SD-4∙4) versus 26∙4 (SD-4∙2) (adjusted mean difference 3∙4 (95%Cl- 2∙12 to 4∙69, p = 0∙001). This pointed out to the improvement in mental health outcomes of the adolescent mothers.  In regard to social outcomes, adolescent mothers in the intervention group performed better on parenting skills at six-month postnatal follow-up compared to mothers in the control group. |
| Kachingwe et al (2021) | Malawi | Provision of safe and inclusive community spaces for adolescent mothers and pregnant girls by Young Women’s Christian Association (YWCA) coordinators and champions (YWCA peer educators).  Psychosocial program by peer educators to build resilience, self-esteem and mutual support among adolescent mothers and pregnant girls. | Nutritional practice  Motors skills and cognitive functions | Parenting skills  Socioemotional capacities | On the part of the intervention group, there was a statistically significant increase in knowledge on parenting skills (p <0.01), nutritional practice (p < 0.01), motor skills and cognitive functions (p < 0.01) as well as expressive language and socioemotional capacities (p < 0.01), while the change in confidence and psychosocial well-being was not significant (p = 0.8823), showing that there was no change. However, there was no significant change for the control group for all parameters. |
| Leerlooijer et al (2013) | Uganda | Teenage mother project (TMP) with five components namely; community sensitization, teenage mothers support groups, livelihood (continued education and income generation), counselling and advocacy. | Mental well-being through improved self-confidence and self-worth and coping skills with adolescent motherhood and stigma. | Community support  Increased agency  Continuation of schooling  Increased earnings among teenagers | A qualitative evaluation to document the changes observed as a result of implementation of TMP revealed that the intervention contributed to overall well-being of unmarried teenage mothers and to a supportive social environment.  The project contributed to supportive community norms towards teenage mothers’ position and future opportunities, increased agency, improved coping with early motherhood and stigma, continued education through returning to school after pregnancy, and increased income generation by teenage mothers.  The study findings also suggest limited change in disapproving community norms regarding out-of-wedlock sex and pregnancy, late active enrolment of teenage mothers in the project (i.e., ten months after delivery of the child), and differences in the extent to which parents provided support. |
| Levy et al (2021) | Kenya | Home visiting program by mentors (who were young mothers aged 19–30 years) | Modern family planning | Household support | A quasi-experimental study capturing before and after comparisons among the same group of non-randomised participants at both time points. The aim was to evaluate home visiting programme to determine its impact on health and social outcome**s** of pregnant adolescent girls, adolescent mothers and their infants.  During the programme, household support increased from 57% to 85%, while 100% of eligible participants were on ART and virally suppressed (total of 20 adolescents).  100% of infants (total of 17 infants) had an up-to-date PCR test with no seroconversions.  Uptake of modern family planning increased from 39% at baseline to 64% at end line (P < 0.001). The referral rate declined from 84% to 78% from baseline to end line with low uptake of referrals for mental health services**.**  (17.3%). |
| Mhlanga et al (2024) | Zimbabwe | Peer-based approach called "Champions of Change," which involved establishing targeted peer support groups for pregnant adolescents and adolescent mothers. | Sexual and reproductive health knowledge | Parental attitudes toward adolescents’ access to sexual and reproductive health services  Health seeking behaviors | Pregnant adolescents and adolescent mothers reported a 43% increase from baseline to endline in accessing adolescent friendly SRHR services, 25% increase in the number of adolescents with a positive attitude towards the use of long-acting reversible contraceptives and 42% increase in the number of adolescents reporting improved attitudes on SRHR among parents. |
| Mwenda et al (2023) | Kenya | Targeted messages on childcare and nurturing and psychosocial support groups for adolescent mothers, moderated by community extension workers and counselors | Not indicated | Maternal knowledge of childcare and infant development milestones and exclusive breastfeeding | Key outcomes were maternal knowledge on childcare and infant development milestones assessed using the Developmental Milestones Checklist (DMC III). Knowledge and DMC III scores were compared between the intervention and control groups, as well as between the two intervention groups.  Compared with the control group, adolescent mothers receiving the full package had a higher knowledge score on infant care and development (9.02 vs 8.01; P<.001) and higher exclusive breastfeeding rates (238/375, 63.5% vs 112/220, 50.9%; P=.004), and their infants had higher average DMC III scores (53.09 vs 48.59; P=.01).  The limited package group also had higher knowledge score than the control group (8.73 vs 8.01; P<.001); this group performed better than the full package group on exclusive breastfeeding (297/416, 71.4% vs 112/220, 50.9%; P<.001) and DMC III scores (58.29 vs 48.59; P<.001) when compared with the control group. |
| Opondo et al (2020) | Kenya | School guidance and counseling program | Not indicated | Social and academic adjustment of teenage mothers | The study adopted ex post facto’s Causal-comparative research design to investigate the Guidance and Counselling Programme and social and academic adjustment of teenage mothers in secondary schools in Kenya.  Student mothers registered the highest rating in social adjustment and they recorded the least rating in academic adjustment.  However, students who had gone through formal guidance and counselling program generally registered higher adjustments than those who did not pass through formal counseling. The teenage mothers’ attitudes had a mediating effect on the relationship between guidance and counseling program and the overall adjustment of teenage mothers. |
| Tinago et al (2024) | Zimbabwe | Community-based peer support program that aimed at mitigating social isolation and stigma among adolescent mothers in Harare, Zimbabwe. | Depressive symptoms and common mental disorders | Household and friends’ support | There was a decrease in the probability of moderate to severe depression among the intervention participants, from 0.408 to 0.231 (p=0.005) and an increase among the control participants 0.267 to 0.333 (p=0.005).  The four scores of measuring the social support from family, friends and significant others had greater improvements in the intervention group compared to control group. |
| Yator et al (2021) | Kenya | Group interpersonal therapy intervention (IPT-G) delivered by community health workers | Depressive symptoms | Social functioning (participants’ ability to maintain interpersonal relationships, social interactions, and fulfill everyday normal social roles.) | A qualitative post-intervention evaluation revealed that PPAs reported notable improvements in social interaction, decision making, and communication skills as a result of the intervention.  At the end of the intervention, some Postpartum adolescents (PPA had established small busine**sses** and displayed improvement in communication and interaction with others peer-to-peer **s**upport. Findings suggest that IPT-G ameliorates depressive symptoms and improves the social functioning of the PPA. |
| Yator et al (2022) | Kenya | Group Interpersonal Psychotherapy (IPT-G) | Depressive symptoms  Antiretroviral therapy adherence | No information provided | A reduction in depressive symptoms after IPT-G administration, with a stronger effect size of 1.57 (95%CI =0.62 to 2.49) in the intervention group than in the control group, with an effect size of 0.65 (95% CI=–0.18 to 1.47). No difference was observed between groups in antiretroviral therapy adherence. This intervention contributed to improvement in mental health outcomes among the postpartum HIV positive young women targeted. |
